# The small RNA diversity from *Medicago truncatula* roots under biotic interactions evidences the environmental plasticity of the miRNAome

**DOI:** 10.1186/s13059-014-0457-4

**Published:** 2014-09-24

**Authors:** Damien Formey, Erika Sallet, Christine Lelandais-Brière, Cécile Ben, Pilar Bustos-Sanmamed, Andreas Niebel, Florian Frugier, Jean Philippe Combier, Frédéric Debellé, Caroline Hartmann, Julie Poulain, Frédérick Gavory, Patrick Wincker, Christophe Roux, Laurent Gentzbittel, Jérôme Gouzy, Martin Crespi

**Affiliations:** Université de Toulouse; UPS; UMR 5546, Laboratoire de Recherche en Sciences Végétales, BP 42617 Auzeville, F-31326 Castanet-Tolosan, France; CNRS; UMR 5546, BP 42617, F-31326 Castanet-Tolosan, France; INRA, Laboratoire des Interactions Plantes-Microorganismes (LIPM), UMR441, Castanet-Tolosan, F-31326 France; CNRS, Laboratoire des Interactions Plantes-Microorganismes (LIPM), UMR2594, Castanet-Tolosan, F-31326 France; ISV (Institut des Sciences du Végétal), CNRS, Saclay Plant Sciences, F-91198 Gif sur Yvette, France; Université Paris Diderot, Sorbonne Paris Cité, F-75205 Paris, France; Université de Toulouse; INP; EcoLab (Laboratoire Ecologie Fonctionnelle et Environnement), ENSAT, 18 chemin de Borde Rouge, 31326 Castanet-Tolosan, France; CNRS-EcoLab (Laboratoire Ecologie Fonctionnelle et Environnement), 31326 Castanet-Tolosan, France; CEA, Genoscope, 2 rue Gaston Crémieux, 91000 Evry, France

## Abstract

**Background:**

Legume roots show a remarkable plasticity to adapt their architecture to biotic and abiotic constraints, including symbiotic interactions. However, global analysis of miRNA regulation in roots is limited, and a global view of the evolution of miRNA-mediated diversification in different ecotypes is lacking.

**Results:**

In the model legume *Medicago truncatula*, we analyze the small RNA transcriptome of roots submitted to symbiotic and pathogenic interactions. Genome mapping and a computational pipeline identify 416 miRNA candidates, including known and novel variants of 78 miRNA families present in miRBase. Stringent criteria of pre-miRNA prediction yield 52 new mtr-miRNAs, including 27 miRtrons. Analyzing miRNA precursor polymorphisms in 26 *M. truncatula* ecotypes identifies higher sequence polymorphism in conserved rather than Medicago-specific miRNA precursors. An average of 19 targets, mainly involved in environmental responses and signalling, is predicted per novel miRNA. We identify miRNAs responsive to bacterial and fungal pathogens or symbionts as well as their related Nod and Myc-LCO symbiotic signals. Network analyses reveal modules of new and conserved co-expressed miRNAs that regulate distinct sets of targets, highlighting potential miRNA-regulated biological pathways relevant to pathogenic and symbiotic interactions.

**Conclusions:**

We identify 52 novel genuine miRNAs and large plasticity of the root miRNAome in response to the environment, and also in response to purified Myc/Nod signaling molecules. The new miRNAs identified and their sequence variation across *M. truncatula* ecotypes may be crucial to understand the adaptation of root growth to the soil environment, notably in the agriculturally important legume crops.

**Electronic supplementary material:**

The online version of this article (doi:10.1186/s13059-014-0457-4) contains supplementary material, which is available to authorized users.

## Background

The root system plays fundamental roles in plants, ranging from anchoring plants in the soil to water and nutrient acquisition as well as interacting with a large variety of rhizospheric organisms. Modulating root growth and branching allows plants to improve these functions [1]. Understanding the molecular mechanisms governing this root developmental plasticity and its adaptation to the soil environment is therefore crucial for crop improvement in sustainable agriculture.

Plants have developed strategies to better acquire nutrients with the help of beneficial soil microorganisms. Around 80% of land plants enter root symbioses with arbuscular mycorrhizal (AM) fungi (the AM symbiosis). In addition, legumes (Fabaceae) are able to form an elaborate symbiosis (the rhizobium-legume (RL) symbiosis) with nitrogen-fixing rhizobacteria to form specialized root organs called nodules. Establishment of AM and RL symbioses requires complex dialogue between the two partners, including the perception by the roots of specific lipo-chitooligosaccharides (LCOs), called Myc-LCO and Nod factors, respectively. As the first components of these signaling pathways are shared, it has been suggested that the specific RL symbiosis may derive from the most ancestral and widespread mycorrhization signaling pathway [2]. Although these two types of beneficial relationships imply very different modifications of roots in the host plant, *that is,* lateral organogenesis of the nitrogen-fixing nodules or formation of arbuscules in cortical cells for endomycorrhization, both promote root growth and function as root morphogens [3].

In contrast to the responses to symbiotic organisms, roots also need to trigger defense responses to soil-borne pathogen attacks. Many pathogenic bacteria and fungi enter the roots and spread rapidly in the plant, inducing typical disease symptoms [4]. Pathogenic and symbiotic relationships are often studied separately and how beneficial microbes may affect host resistance to pathogens and vice versa is still debated [2]. Protective effects of AM symbiosis against pathogens and parasitic plants have been described for many plant species, including agriculturally important crops [5], whereas altered responses of RL symbiotic mutants to various pathogens highlighted putative crosstalk between RL symbiosis and defense pathways [6,7]. Indeed, pathogenic and symbiotic interactions both involve early defense reactions involving chitin or associated LCO chitin-related symbiotic signals [8]. Hence, signaling pathways that mediate root symbiotic and pathogenic relationships may be interconnected and differential regulation of defense responses can be critical for the establishment of successful symbiotic interactions.

Small non-coding RNAs (smRNA) have emerged as key players in many signaling pathways that control development and responses to the environment in eukaryotes. In plants, smRNAs are mainly 20 to 24 nucleotides in length and are divided into microRNAs (miRNA) and short-interfering RNAs (siRNAs). miRNAs, mainly 20 to 22 nucleotides in length, are processed from miRNA precursors folded into an imperfect stem-loop secondary structure by a DICER-LIKE protein called DCL1. The DCL1-mediated slicing of the hairpin precursor produces a small double-stranded RNA with two-nucleotide 3’ overhangs, called the miR:miR* duplex. After loading of one strand into an effector RISC complex, the miRNA binds a target RNA by base-pairing, leading either to its cleavage or to inhibition of its translation. Other smRNAs are produced from long double-stranded RNAs, generated either by antisense complementary transcripts or through the action of plant-specific RNA-dependent RNA polymerases. These siRNAs can repress the expression of target genes through post-transcriptional or transcriptional regulation [9]. Several miRNAs, conserved in most angiosperms, have been linked to the control of root architecture. Many of them, like miR160, miR164, miR167, miR390 and miR393, directly or indirectly regulate genes related to auxin signaling [10]. In addition, the mobile miR165/166, together with its HD-ZIP transcription factor (TF) targets, is at the heart of a subtle cellular communication, regulating radial patterning of the root vasculature, pericycle and endodermis (reviewed in [11]). Other miRNAs, such as miR395 and miR399, are involved in adaptive responses to local variations in nutrient availability (sulfate and phosphate, respectively) in the soil [10]. Most of these studies were performed in *Arabidopsis thaliana,* which is not able to establish root symbioses with either AM fungi or rhizobia [10]. In legumes, a role of miR399 in responses to increased phosphate, a nutritional status linked to effective mycorrhization, was reported by Branscheid *et al.* [12]. Other miRNAs, like miR164, miR166 or miR396, were shown to play indirect roles in nodule development or mycorrhizal symbiosis due to their global impact on auxin responses and/or tissue patterning in roots [13–15]. Concerning plant-pathogen interactions, miRNAs linked to auxin signaling were related to defense reactions in *A. thaliana* [16,17] and more recent studies provided evidence that some miRNAs function as master regulators of disease-resistance genes encoding nucleotide-binding site leucine-rich repeat (NBS-LRR) proteins in diverse plants [18,19]. In legumes, other miRNAs play specific roles in nodulation. For instance, in *Medicago truncatula,* miR169a controls nodule meristem maintenance through the repression of *HAP2*, which encodes a nodulation-responsive TF [20]. In soybean, over-expression of three miRNAs, gma-miR482, gma-1512 and gma-1515, specifically increased nodule numbers without affecting root development *per se* [21]. Recently, De Luis *et al.* [22] and Lauressergues *et al.* [23] showed that specific variants of miR171 control RL symbiosis in *Lotus japonicus* and AM fungal colonization in *M. truncatula*, respectively*.* Instead of the conserved *SCARECROW-like* GRAS TF targets of miR171, these miR171 variants recognize a different but related *NSP2* GRAS TF involved in molecular regulation associated with the common symbiosis (sym) pathway [22–24]*.* Hence, the evolution of miRNA regulation led to novel gene expression patterns in diverse root developmental mechanisms.

In legumes, two wild species, barrel medic (*M. truncatula)* and *L. japonicus*, and the cultivated soybean (*Glycine max*) have been adopted as models for genomic studies. For *M. truncatula*, genome sequencing data [25,26] (see Materials and methods) and very large expressed sequence tag collections (TIGR v.11.0, March 2011) are available. miRBase (v.20, June 2013) includes more than 5,100 precursors of miRNAs from 67 plant species and lists more than 200 families for *M. truncatula* and soybean, coming from different tissues and/or treatments, but only three for *L. japonicus*. Yet, De Luis *et al.* [22] recently identified 35 novel miRNAs in *Lotus*, in addition to the well-known conserved families in angiosperms. There are also some mis-annotations as re-evaluation of rice miRNAs with recently updated miRNA criteria revealed that around 150 are likely siRNAs [27].

In this study, we present global analyses of deep sequencing data from 20 smRNA libraries of *M. truncatula* roots, grown under different pathogenic or symbiotic interactions as well as treated with Myc-LCO/Nod factors. Genome-wide identification of miRNAs and statistical comparisons between libraries revealed both large diversity and plasticity of the root miRNAome. Conservation and evolution of mtr-miRNA precursor genes were addressed through sequence comparison among several angiosperms and in 26 *M. truncatula* genotypes. These results highlight the potential role of miRNAs in diverse adaptations of legume root growth to a variety of soil environments.

## Results and discussion

### Genome-wide identification of miRNA candidates

**Figure 1:**
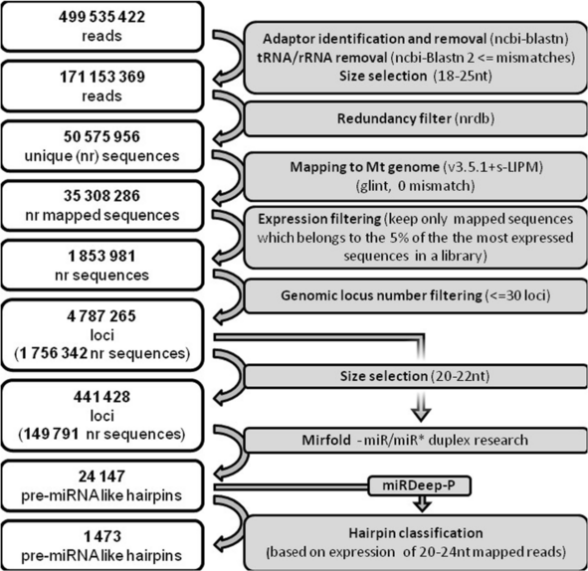
**Pipeline used for** ***M. truncatula*** **miRNA identification.** Counts for reads, unique/non-redundant (nr) sequences, genomic loci detected or pre-miRNA-like hairpins are given at each step of the pipeline (white boxes). Details about each filtering step (grey boxes) are indicated in Materials and methods. The arrows represent the direction of the pipeline. Mt, *Medicago truncatula*; nt, nucleotide; wgs, whole genome shotgun.

To investigate miRNA diversity in *M. truncatula*, we constructed smRNA libraries from *M. truncatula* roots and root tips grown under control conditions or submitted to diverse fungal or bacterial pathogenic and symbiotic interactions (Additional file 1). Sequencing of smRNA libraries using Solexa technology (Illumina) provided between 4,524,240 and 33,510,667 reads for each condition, corresponding to a total of 50,575,956 non-redundant (nr) RNAs from 18 to 25 nucleotides in length after removal of rRNA and tRNA-associated sequences (Figure 1). For this set of smRNAs, genome-wide identification of putative miRNA genes was performed on the *M. truncatula* genome, using a pipeline adapted from Lelandais-Brière *et al.* [28] (Figure 1). Out of 35,308,286 nr smRNAs with perfect matches on the genome and after removal of smRNAs of very low abundance (see Materials and methods), a total of 1,853,981 smRNAs were retained (Figure 1). We also eliminated around 100,000 mapped smRNAs, with more than 30 genomic loci probably corresponding to repeat associated-siRNAs. For the 1,756,342 remaining nr smRNAs, prediction of pre-miRNA-like secondary structures was performed following criteria from Meyers *et al.* [29]. This resulted in 24,157 pre-miRNA like secondary structures, which were classified into 5 categories mainly according to their smRNA distribution profiles (see Materials and methods). Class 1 and 2 hairpins (class 1 are stem-loops producing only miRNA and miR*, whereas class 2 are stem loops producing other rare additional smRNAs; see Materials and methods) can be considered as pre-miRNA candidates, as both miRNA and miR* sequences were found in our data set, a criterion strictly required for certification of plant miRNAs [29]. In addition, these precursors produced at least 10-fold more annotated miRNAs than any other additional smRNA, as commonly observed for miRNA genes. We also used miRDeep-P [30] to select and classify miRNAs (Materials and methods; Figure 1). Sets of 542 and 71 pre-miRNA genes fall into classes 1 and 2, respectively, and 159 pre-miRNA genes from classes 3, 4 and 5, producing similar mature miRNAs to genes from classes 1 and 2, were added [31]. In total, these 772 loci encode 416 nr mature smRNAs (Additional file 2). More than 36% (282) of these precursors have been identified thanks to the novel 140 Mbp of genomic sequences disclosed by our study.

As expected, most predicted miRNAs had a 5’ uridine residue (62%) and were 21 nucleotides in length (92%). However, this percentage is biased due to our selection procedure. Indeed, when several isoforms of 20, 21 or 22 nucleotides matched perfectly inside the miR-miR* region of a precursor, the 21-nucleotide form was chosen as the 'defining smRNA'. Recently, Zhai *et al.* [32] reported that 22-nucleotide variants of certain miRNA families (*that is,* miR1507, miR1509, miR2109, miR2118 and miR2597) accumulate at higher levels than the 21-nucleotide form in *M. truncatula* and soybean. In our analysis, we found 63 such miRNAs (15.1%), including certain miRNAs described by Zhai *et al.* [32], except for miR1509, which was absent from our sequencing. Thus, we expanded the set of 22-nucleotide miRNAs in the *M. truncatula* legume (Additional file 2).

**Figure 2:**
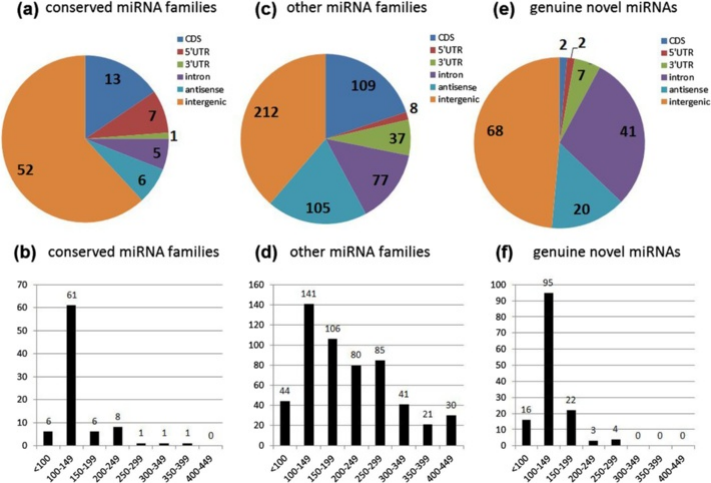
**Genomic annotation and size distribution of the predicted pre-miRNAs. (a-f)** Pie charts represent the distribution of the pre-miRNA genomic loci on the *M. truncatula* genome for the conserved miRNA families **(a)**, other known miRNA families **(c)**, or the 52 genuine novel miRNAs **(e)**. Numbers of miRNAs present in coding sequences (CDS), antisense to CDS, intergenic regions, and 5’ UTR or 3’ UTR regions of annotated transcripts are shown. Histograms represent the size distribution of the pre-miRNAs for the conserved miRNA families **(b)**, other miRNA families **(d)** or the 52 genuine novel miRNAs **(f)**. Numbers of miRNAs present in each size class are shown.

To account for variant diversity inside miRNA families, smRNAs with less than four sequence mismatches were grouped. Taken together, the 416 selected candidates fitted into 365 families (Additional file 2), corresponding to a total of 772 putative genes (Additional files 3 and 4). Predicted pre-miRNA length varied from 58 to 439 nucleotides, with an average of 187 nucleotides, and 79.3% comprised between 100 and 300 nucleotides (Figure 2b). This is consistent with reports in other species (*for example,* [33]). Complete lists and the main characteristics of miRNAs and their precursors - dG, length, miRDeep-P score, coordinates and annotation in the genome - are given in Additional file 3 for miRNAs found in intergenic regions, and Additional file 4 for precursors matching to loci annotated as intragenic. As expected for miRNA genes (Figure 2a,c,e), most were found in intergenic regions (332 hairpins, 43%) or antisense to other genes (131 hairpins, 17%). However, around 16% mapped to introns (123 hairpins), 8% to untranslated regions (62 hairpins) and 15% to predicted coding sequence (CDS; 124 hairpins).

Therefore, we provide a curated, expert-based database for legume miRNAs, with precise and accurate genome locations that, to our knowledge, constitutes the most expansive effort to characterize the root miRNAome under a variety of symbiotic and pathogenic interactions and related signals.

### Characterization of known mtr-miRNA families

In miRBase (v.20), 263 miRNA families (672 genes) are listed for *M. truncatula*. Twenty-one mtr-miRNA families (according to the miRBase nomenclature 'mtr-XXX') correspond to the so-called 'conserved' miRNAs, which are found in most angiosperms [34]. The remaining 242 families mainly arose from smRNA libraries [28,32,35,36] in organs (leaves, stems, flowers, seeds, roots) or under symbiotic interactions (roots inoculated with *Sinorhizobium meliloti* or the AM fungus *Rhizophagus irregularis*). BlastN on miRBase revealed that 125 of our candidates corresponded to members of these known mtr-miRNA families, including 65 identical to already registered mature miRNAs or miR* (green highlighted entries in Additional file 2) and 60 novel variants of them (yellow highlighted entries in Additional file 2). These miRNAs fitted into 78 families, *for example,* 21 'conserved' (miR156 to miR530) and 57 others (miR1507 to miR7696). The size distribution of the precursor and annotation of conserved miRNAs are shown in Figure 2a,b and, as expected, these miRNAs are mainly encoded in intergenic regions.

In the published version of the *M. truncatula* genome, Young *et al.* [25] annotated 196 previously reported mtr-miRNAs. These authors discussed that miRBase-registered miRNAs lacking in their study may either display specific expression patterns or were not genuine miRNAs. Additional smRNA sequencing from *M. truncatula* seedlings treated with mercury [37] and roots treated with ethylene or aluminum [38,39] allowed the identification of 52, 20 and 3 novel families, respectively, which were not listed in miRBase. Our analysis was focused on roots, which probably represent only a fraction of miRNA diversity, and certain known miRNAs might be present in these data but have not been retained, due to low abundance or because no miR* was found in the libraries. The presence of 20 well-known conserved miRNA genes in classes 3, 4 or even 5 reinforces this hypothesis. Finally, in most previous reports (*for example,* [28] or [36]), the presence of the miR* was not considered as an absolute prerequisite, and some registered mtr-miRNAs in miRBase are likely to be false candidates. In particular, this may be the case for miR2630, which we identified previously [28] but rejected from the present study due to the absence of miR*.

It was generally assumed that the mature miRNA (or guide miRNA) accumulates at higher levels than the miR* (or passenger miRNA), which may not be loaded into the RISC complex. However, Devers *et al.* [36], *for example,* recently showed in legumes that the miR* may accumulate at similar or even higher levels than the miRNA in particular conditions or tissues. In our analysis, only four passenger strand sequences of the conserved miR393, miR1507, miR2118 and miR7696 families were found in higher counts than their corresponding guide miRNAs (ptc-miR393a-3p, 204DVAAXX:1:47:672:376, 204DVAAXX:1:4:984:557 and mtr-miR7696b-3p, respectively; highlighted in pink in Additional file 2). In our pipeline, we annotated as miRNA the most abundant smRNA mapping on a precursor in each condition. However, as we pooled the reads obtained in all libraries, we cannot rule out that some miR* may be more abundant than the corresponding miRNA in particular samples.

Among the conserved miRNAs, we found two genes for miR394 (Additional file 2) not previously reported in *M. truncatula*. In contrast, we did not identify any miR397 family member, an absence already noticed in *M. truncatula* [25,32,36,40]. However, Jagadeeswaran *et al.* [41] detected this conserved miRNA by RNA blot analysis in *M. truncatula* seedlings submitted to prolonged copper starvation, and more recently, Eyles *et al.* [42] reported its presence in *M. truncatula* calli. We thus searched for miR397 reads in our complete set of nr smRNAs and found a 21-nucleotide smRNA (ath-miR397a) identical to gma-miR397a whose accumulation was relatively low (24 reads maximum in one condition). This low accumulation was unexpected because, in other legumes, miR397 was relatively abundant and has been linked to root pathogenic and symbiotic interactions [43]. In addition, De Luis *et al.* [22] reported that this miRNA was systemically induced in nitrogen-fixing nodules of *L. japonicus* and may be involved in the maintenance of copper homeostasis during nodulation. These authors even suggested that miR397 may serve as a systemic marker for the presence of functional nodules in *L. japonicus*. One possibility is that miR397 may be linked to different types of nodules formed in soybean and Lotus (determinate nodules without a persistent meristem) compared with *Medicago* spp. (indeterminate with a persistent meristem).

### Discovery of 52 genuine novel miRNAs in *M. truncatula*

Out of the 416 selected mature miRNA candidates, 291 were novel (*that is,* no homolog with 3 or fewer mismatches was found in miRBase). To select the best genuine miRNAs and distinguish them from potential structured precursors leading to siRNA production, we applied additional selection criteria. First, we decided to eliminate the novel candidates for which less than 20% of their genomic loci had a pre-miRNA stem-loop secondary structure, which most probably correspond to siRNAs. As a control, none of the conserved miRNAs belonging to the 22 families was rejected by this filter. In contrast, known non-conserved families (like miR2592, miR2610, miR2619, miR2645, miR5205, miR5241, miR5283, miR5287, miR2590) but also 73 novel candidates were discarded (grey lines in Additional file 2: Table S2a,c). Second, all precursors were reanalyzed using miRDeep-P, a software commonly used for plant miRNA identification, and only smRNAs with at least one precursor accepted by miRDeep-P were considered (Figure 1). This allowed the selection of a restricted final set of 52 novel miRNA candidates, which fulfill all criteria for being genuine miRNAs (Additional file 5 and details in Additional file 2: Table S2b).

As expected, at least one precursor of each novel mtr-miRNA family was located outside an annotated transposable element, a repetitive element or an exon (Additional file 5). Indeed, 15 genes lie in intergenic regions, 3 are antisense to a protein-coding gene and 34 reside in annotated untranslated regions (7) or introns (27) (Figure 2e,f). Introns can host small nucleolar RNA, miRNA or even long non-coding RNA genes. In human, half of the miRNA precursors reside within introns and are co-expressed with their host gene [44]. In plants, however, only 1, 10 and 5 so-called 'miRtrons' have been reported in *M. truncatula*, *A. thaliana* and rice, respectively [28,45,46]. Thus, the high number of putative miRtrons in these candidates was unexpected. Even if accumulation was detected, however, we cannot rule out that some of the predicted miRtrons may in fact reside in incorrectly annotated intergenic regions or are generated by intergenic precursors present in as yet unsequenced areas of the genome. To validate their accumulation, we performed quantitative RT-PCR analyses for 35 novel miRNAs, including 3 miRtrons, in several independent RNA samples corresponding to roots, symbiotic interactions and pathogenic interactions (Additional file 6: Figure S1).

### Complexity of mtr-miRNA gene families

Most miRNA families identified in this study (339/365 families, 92.9%), including 97% of the novel ones, corresponded to a unique mature miRNA variant in our samples (Additional file 6: Figure S2; Additional file 2). The large proportion of *M. truncatula-*specific miRNA families may be explained by neutral evolution of these newly spawned miRNAs [47]. This neutral evolution is probably due to the fact that most newly evolved miRNAs do not play any role or, if they do, they are not essential roles in regulatory networks, and genes encoding such miRNAs may then spawn and disappear at a high frequency [47]. In fact, few families contained more than five variants and the greatest complexity was found in three conserved families (Additional file 6: Figure S2; Additional file 2: Table S2a), miR156 and miR169 (6 variants each) as well as miR171 (7 variants). It is interesting to note that specific roles and/or targets had already been reported for certain variants of these families. For example, Naya *et al.* [48] showed that, in addition to the well conserved squamosa binding protein targets, a novel mtr-miR156 isoform was able to cleave a transcript coding for a WD40-like protein in *M. truncatula* root tips. In addition, lja-miR171c and mtr-miR171h specifically regulate *NSP2*, which encodes a key GRAS TF involved in both RL and AM symbioses [22-24]. These miR171 variants thus may have coevolved with *NSP2* genes in plants undergoing endosymbioses. In contrast to conserved miRNAs, the roles of novel miRNAs remain poorly investigated. In soybean, the miRNA miR1515 was linked to the control of nodule number [21]. This miRNA was able to cleave two transcripts coding for a GSK3-like kinase and a DICER-like 2 protein. Although more functional studies are needed, novel miRNA-related pathways in legumes may thus be attributed both to the diversification of preexisting miRNA families and their targets and to the emergence of new miRNAs.

Taking into account their different variants, between 1 and 64 genes were identified per miRNA family (Additional file 2). However, the large majority (including most novel miRNAs) were monogenic (259/365 families, 71%). Only 3 families contained more than 15 predicted hairpins (miR2111, 18 genes; miR2592, 64 genes; and AZOTE_0001_61FVGAAXX:1:10:1676:21066, 23 genes). For known families, gene numbers are generally identical or very similar (±2 genes) to those from miRBase. Interestingly, three additional miR167 precursors and a novel variant were found, suggesting a more complex regulatory module than previously reported for this auxin-related miRNA [28]. In contrast, our procedure clearly led to an underestimation of certain families, in particular miR169, miR395 and miR399. Indeed, for these families, we identified 7, 11 and 13 precursors, respectively, instead of the 18 genes per family already listed in miRBase. The variants produced by the missing precursors may not accumulate at sufficient levels in the root samples analyzed, or the miR* was not detected.

### mtr-miRNA conservation among angiosperms

**Figure 3:**
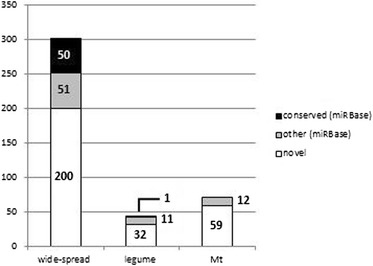
**Distribution of the mature miRNAs in different species.** Sequences found widespread (based on analyses of six plant genomes; see Materials and methods), in only *L. japonicus*, *M. truncatula* and *G. max* legumes, or in only *M. truncatula* were identified. Boxes are proportional to the number of miRNAs in each category (shown in the bars). Mt, *M. truncatula.*

To evaluate miRNA conservation in angiosperms, we searched for homologues of the 416 candidates in genomes of eight other species: three Fabaceae (*L. japonicus, Phaseolus vulgaris* and *G. max*), three non-legume eudicots (*Vitis vinifera*, *Populus trichocarpa* and *A. thaliana*) and two monocots (*Oryza sativa* and *Zea mays*). Three levels of conservation were considered: 1) wide-spread (not only found in Fabaceae); 2) legume-specific (found only in Fabaceae); and 3) *M. truncatula*-specific. In addition to 101 miRNAs from already known families, 200 novel candidates fitted into category 1 (Figure 3), thus revealing a significantly high proportion of novel miRNAs already present in other plants. Among these, however, only 8 were found in all angiosperm families tested and 14 appeared to be specific to eudicots (Additional file 6: Figure S3). Out of the 44 legume-specific miRNAs identified in category 2 (Figure 3), 6 are common to the three model legumes, including 5 novel ones. Finally, 71 (59 new) smRNAs were specific to *M. truncatula* (Figure 3). Interestingly, among the legume-specific miRNAs, two isoforms of miR169 (mtr-miR169p and mtr-miR169q) clearly diverged from the conserved mature variant (Additional file 2: Table S2a). Combier *et al.* [20] reported that mtr-miR169a over-expression led to the repression of the nodule-specific *MtHAP2-1* TF in *M. truncatula* nodules and that the regulation of *MtHAP2-1* by this miRNA may be essential for the differentiation of nodule cells. However, mtr-miR169a accumulated at very low levels in smRNA libraries and its unique precursor was classified in class 3 due to the absence of a miR*; thus, it was filtered out by miRDeep. Six other variants of the miR169 family (Additional file 2: Table S2a) were identified in our process, suggesting that, despite the effect of mtr-miR169a ectopic over-expression, putative specialization/increased efficiency of the miR169 variants in legumes may have occurred.

In *A. thaliana*, 70% of the reported miRNAs are specific to Brassicaceae [47]. In 2010, the availability of two genomes from *Arabidopsis* species, *A. thaliana* and *A. lyrata*, allowed comparison of the miRNA populations between these sister species [49]. The miRNAs identified were divided into three categories: 1) 'deeply conserved' miRNAs, corresponding to our 'wide-spread miRNA' category, with loci in non-brassicaceae species; 2) 'less conserved' miRNAs with only Brassicaceae miRNAs, corresponding to our 'legume-specific' category; and 3) 'non-conserved' miRNAs specific to only one of the *Arabidopsis* species, equivalent to our '*M. truncatula*-specific' miRNAs. They found 104, 38 and 78 miRNAs in each of these categories, respectively. An equivalent partitioning between the categories was found for *Medicago* miRNAs (200, 44 and 71, respectively, for each category), with slightly more miRNAs found in the conserved families compared with legume-specific or *M. truncatula-*specific miRNAs. This result suggests that we probably identified new miRNAs conserved among several angiosperm species. The deep sequencing approaches have not yet reached a point of exhaustiveness and allow, even today in highly studied species, identification of not only species-specific miRNAs but also deeply conserved miRNAs with interaction-, spatial- or time-specific expression profiles. These predicted conserved miRNAs are particularly interesting for understanding the evolution of miRNA functions among angiosperms.

### Conserved miRNA genes show more polymorphism than specific miRNAs in 26 accessions of *M. truncatula*

Based on whole-genome resequencing data available for 26 *M. truncatula* accessions [50], we investigated sequence polymorphisms in the 772 miRNA genes identified (corresponding to the 416 mature miRNA candidates). We obtained SNP data for 529 genes because some genes were not present in the HapMap (haplotype map) *M. truncatula* sequence data [50]. Based on a set of 2,316,489 SNPs, we then surveyed the variation in the mature miRNAs, in the hairpin regions (*that is,* putative pre-miRNAs) and the 1,500-bp upstream and downstream flanking regions of the pre-miRNAs, which may be part of the pri-miRNA and/or contain introns as well as regulatory sequences of the putative miRNA genes.

Only a very small fraction of the mature miRNAs had SNPs at position 10 or 11 in the mature miRNA, with no significant differences between known and novel miRNAs (1.5% and 1.9%, respectively; Table 1). No significant difference in SNP abundance was observed when comparing the 1 to 7, 8 to 12 and 13 to 21 positions within the mature miRNA. As already observed in *A. thaliana* [51], the reduced level of sequence variation in mature miRNAs suggests that strong purifying selection has acted on them, even on evolutionarily young ones, indicating the newly discovered miRNAs have effective biological roles.

**Table 1:** **SNP distribution among the** ***M. truncatula*** **chromosomes**

		**Number of precursors**			**Precursors with at least one SNP**			**Precursors with at least one SNP in mature miRNA**			**Precursors with a SNP at position 10 or 11 in mature miRNA**		
	**SNP number**	**Total**	**Conserved**	**Novel**	**Total**	**Conserved**	**Novel**	**Total**	**Conserved**	**Novel**	**Total**	**Conserved**	**Others**
Chr 1	198,291	27	9	18	11	4	7	2	0	2	0		
Chr 2	266,911	54	5	49	28	4	24	6	0	6	2		2
Chr 3	317,117	61	7	54	24	7	17	8	0	8	0		
Chr 4	302,215	78	7	71	32	5	27	14	0	14	3		3
Chr 5	462,047	92	18	74	44	12	32	12	3	9	2	1	1
Chr 6	140,777	45	2	43	16	1	15	7	0	7	0		
Chr 7	312,760	82	7	75	49	3	46	19	1	18	3		3
Chr 8	227,898	46	6	40	21	4	17	6	0	6	0		
Chr 0	88,473	44	5	39	6	2	4	2	2	0	0		
Total	2,316,489	529	66	463	231	42	189	76	6	70	10	1	9
						Percentage of precursors			Percentage of precursors			Percentage of precursors	
						63.6	40.8		9.1	15.1		1.5	1.9
									Percentage of polymorphic precursors			Percentage of polymorphic precursors	
									14.3	37.0		16.7	12.9

First column indicates the chromosome number. For each chromosome, the total number of SNPs, precursors, precursors with at least one SNP, precursors with at least one SNP in the mature miRNA sequence and precursors with a SNP at position 10 or 11 in mature miRNA are indicated for the conserved or novel miRNAs. Below, the percentage of precursors in each category is shown.

**Figure 4:**
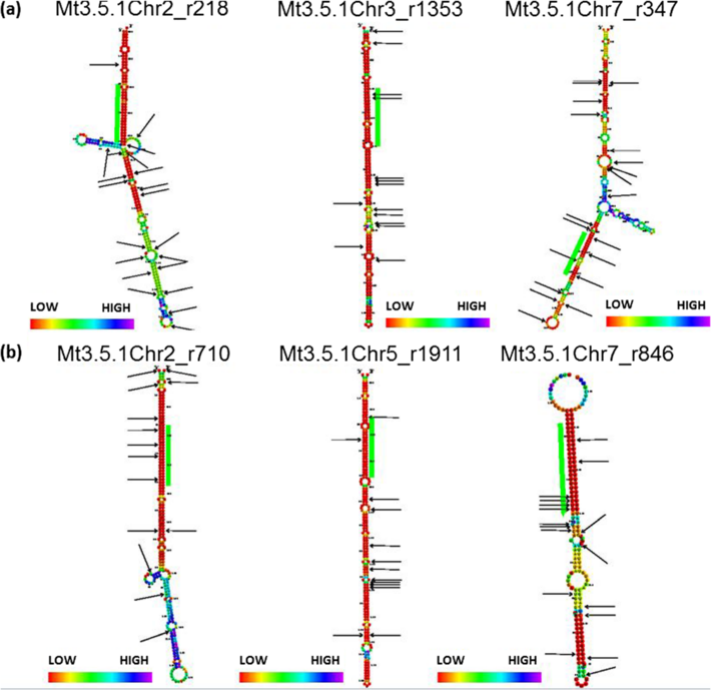
**SNP positions in polymorphic precursors. (a,b)** Examples of conserved miRNAs **(a)** and novel miRNAs **(b)** with SNPs in pre-miRNA hairpins. The ID of the precursor is indicated above the corresponding model. Colors indicate the entropy of the sequence from red (low) to purple (high). The green bars represent the mature miRNA location. Arrows indicate SNP positions on each precursor.

Much less is known about the evolution of pre-miRNAs and flanking regions, in particular for wild species. Among the pre-miRNAs, 231 precursors (44%) had SNPs or small (<2 bp) insertions/deletions (indels) within their sequence in at least one accession (Table 1, Figure 4). It is noticeable that polymorphic precursors of conserved miRNAs are significantly more abundant than those of novel miRNAs, as around 64% of the former contain at least one SNP (Additional file 7). Additional file 7 summarizes polymorphism data for the 529 pre-miRNAs. A precursor of mtr-miR169d-5p, Mt3.5.1Chr2_r218, exhibited as many as 21 SNPs in the hairpin sequence. The Mt3.5.1Chr4_r2510 putative pre-miRNA of the novel miRNA 204DVAAXX:1:294:247:201 had 26 SNPs.

We then searched for particular polymorphic positions on the pre-miRNA molecule. SNP positions are not evenly distributed over the pre-miRNA sequences, as shown in Figure 4a,b for three polymorphic conserved and novel pre-miRNAs, respectively. The large majority (76%) of SNP positions are located in bulges of the molecules or in the immediate vicinity (one or two nucleotides) of them. Allen *et al.* [52] showed that miRNA genes could arise from genes with inverted repeats that may then become a target of a miRNA through evolution of the hairpin [53]. Loci of young miRNAs might have to ‘adapt’ the canonical DCL1-dependent pathway that operates on imperfect miRNA hairpins by accumulating mutations on them [54].

SNP positions in putative miR* sequences are not rare (8%). Todesco *et al.* [55] demonstrated that a single base pair substitution in the miR* sequence of ath-miR164a altered the predicted stability of the miR:miR* duplex, reduced miRNA accumulation and affected leaf shape and shoot architecture in *A. thaliana*. Many other SNPs modified miR:miR* pairing in the same species. We identified dozens of miR:miR* duplexes with a high number of natural polymorphisms that can affect base pairing and thus reduce accumulation of mature miRNAs (examples include Mt3.5.1Chr7_r347 and Mt3.5.1Chr2_r710 in Figure 4).

**Figure 5:**
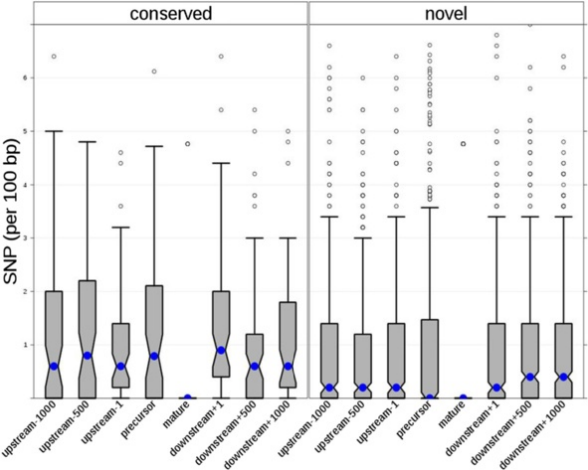
**Distribution of polymorphisms in miRNA genes and surrounding regions from 26 ecotypes of** ***M. truncatula.*** Box plots of the number of SNPs (expressed as SNP/100 bp) at *M. truncatula* miRNA loci, as a function of their location with respect to the pre-miRNA. Data for conserved and novel miRNAs are displayed. Upstream-1000 corresponds to the upstream region from -1,500 to -1,000; upstream-500 corresponds to the upstream region from -999 to -500 and upstream-1 corresponds to the upstream region from -499 to -1. The same nomenclature applies for downstream regions. Precursor stands for pre-miRNA stem-loops and mature stands for 21 or 22 nucleotide miRNA. The blue point indicates the median of SNP abundances. The grey boxes contain 50% of the data and notches indicate the median confidence interval. Whiskers indicate the upper and lower quartiles and no whisker is visible if the lower quartile is equal to the minimum (in that case, the grey boxe contains 75% of the data). Outliers (more than 1.5 interquartile range) are depicted by open circles.

A survey of genetic variations in the 1,500-bp upstream and downstream flanking regions revealed contrasting situations for conserved and novel miRNAs (Figure 5; Additional file 8), a point not addressed in previous studies. Polymorphism is significantly greater for upstream and downstream flanking regions of conserved pre-miRNAs genes: 61/66 (92.4%) of them exhibit SNPs in either pre-miRNA or flanking regions, whereas only 359/463 (77.5%) of the novel pre-miRNAs harbor genetic variability for these regions. We then investigated SNP abundances in consecutive upstream or downstream 500-bp windows (Figure 5). Overall, polymorphisms in pre-miRNAs (*P-value* = 1.3 × 10^-3^) and flanking regions (*P-value* = 8 × 10^-3^) are more numerous in conserved miRNAs than in young ones independently of genomic location (no significant differences in SNP number regardless of whether the precursor was embedded within introns, 5’ UTR, 3’ UTR, CDS, or intergenic regions; *P-value* = 0.1548). This result enabled us to hypothesize that the high levels of polymorphisms in precursors of conserved miRNAs may be due to balancing selection, which will maintain (and accumulate) different alleles over long evolutionary times. This difference between conserved and young miRNAs is not related to the number of copies of conserved miRNA genes, as pre-miRNAs encoded by at most two genes and those encoded by at least three genes exhibit the same amount of SNPs (*P* = 0.16; Additional file 6: Figure S4). For young pre-miRNAs, the range of polymorphisms present in the hairpin is more important than for the other regions (Figure 5), although not significantly different from that of upstream or downstream regions, suggesting that the hairpin region of novel pre-miRNAs is a site of ongoing accumulation of polymorphisms.

Altogether, we hypothesized that the large proportion of SNPs found in miRNA precursors, in particular of conserved miRNAs, may correspond to adaptive driving forces acting during ecotype evolution and adaptation to different environments.

### Many targets of novel mtr-miRNAs are involved in stress responses and signal transduction

To relate mtr-miRNAs to putative functional pathways, we predicted their targets using miRanda [56] and RNAplex [57] and considered the degradome reads obtained by Devers *et al.* [36] and Zhou *et al.* [37] using Cleaveland [58]. In the degradome data, we found up to 24 targets, with an average of 6, per conserved miRNA and only 1 for other known miRNAs (Additional file 2: Table S2a). Thanks to the novel 140 Mbp of genomic sequences and the associated annotations provided by this study, we were able to predict 1,068 of the 3,349 present targets. Target confirmation was not very effective for the novel miRNAs identified (Additional file 2: Table S2b). Indeed, in the case of lowly expressed miRNAs, targets may become more difficult to capture from degradome data [36,37]. Nevertheless, three of the genuine novel miRNAs have a degradome-validated target. E4D3Z3Y01BW0TQ has a corresponding cleavage site in a transcript encoding a gibberellin-regulated protein. This hormone is known to play a crucial role during mycorrhization [59] and this novel miRNA seems to be up-regulated four-fold in mycorrhized plants. Target prediction using miRanda gave a broader spectrum with an average of 19 candidates per novel miRNA. Furthermore, 35 of our newly identified microRNAs, including 6 genuine ones, exhibit an anti-correlated expression with their identified targets in different samples of the *Medicago truncatula* Gene Expression Atlas (MtGEA; Additional file 9). For 22 miRNAs, however, no target was predicted in the genome, whatever the software used. The corresponding miRNA may not target any predicted transcript, their target site may overlap two exons, or the genomic locus of the potential target may not be sequenced yet. The absence of predicted targets for young miRNAs in degradome data was also reported for other species [60,61]. Alternatively, we cannot discard that young mtr-miRNAs may not regulate any target or act at the translational rather than the mRNA cleavage level.

The large majority of conserved miRNA targets (361/380, 95%) have regulatory functions (Additional file 6: Figure S5a), showing pleiotropic effects [61]. Most predicted targets of the legume miRNAs are also involved in these functions (77/143 targets, 53.8%), but also belong to other functional categories, such as proteins with 'catalytic activity' (8.4%) or involved in 'stress responses' (4.2%; Additional file 6: Figure S5b) [62]. The predicted targets for the miRNAs potentially specific to *M. truncatula* show roughly the same distribution as the legume-specific miRNAs (Additional file 6: Figure S5c). In particular, 17% of the predicted *M. truncatula*-specific miRNA targets are involved in signaling (kinases, transferases, receptor activity and signal transducer) and responses to stress. One can imagine that these young miRNAs are involved in regulating appropriate responses to stresses specific to the rhizospheric environments of *M. truncatula,* such as interactions with particular microorganisms (*for example,* symbiotic or pathogenic).

### Towards a global view of miRNA diversity in roots under biotic interactions

**Figure 6:**
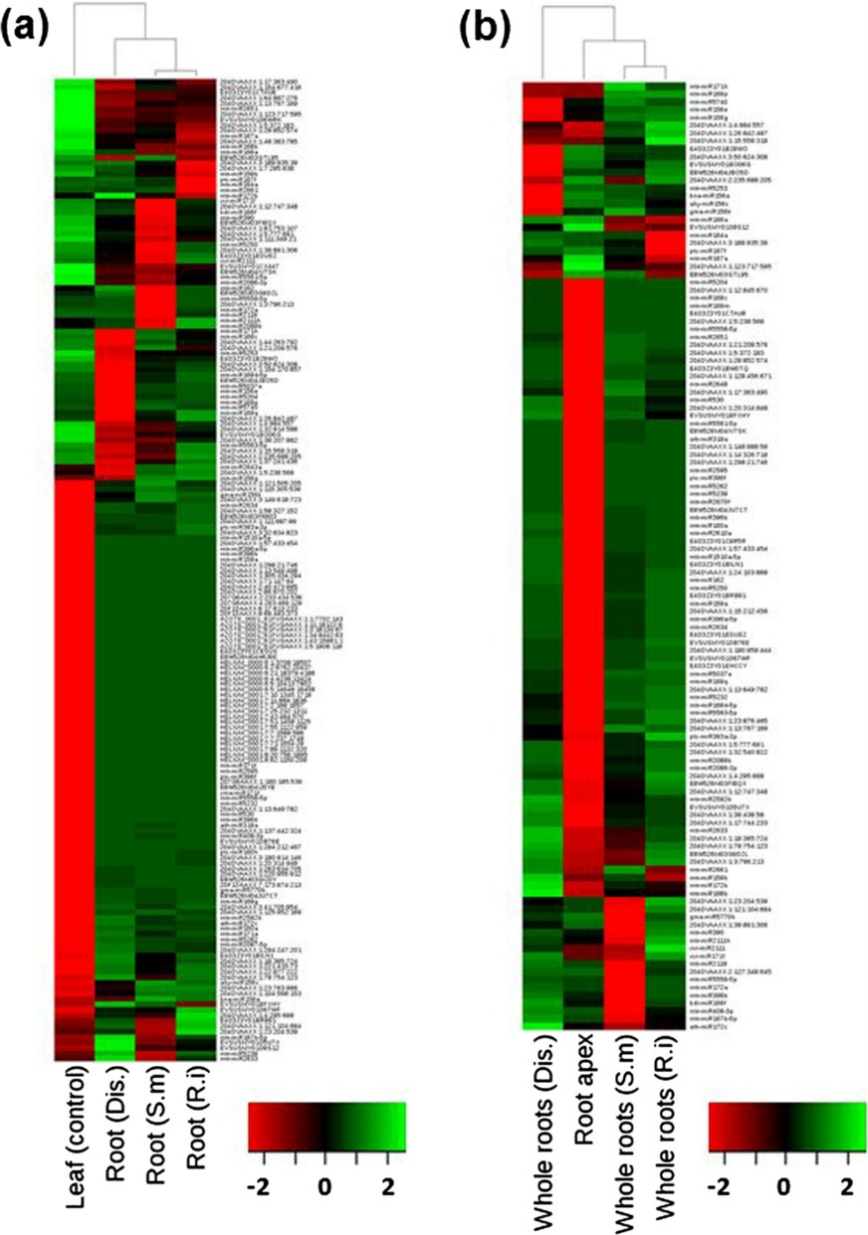
**Heatmaps of differential** **ly expressed miRNAs from different organs of** ***M. truncatula.*** **(a,b)** Heat maps from root libraries *versus* leaf library **(a)** and root libraries *versus* root apex libraries **(b)**. Each column corresponds to the different libraries. miRNA IDs are indicated on the right of the diagram and each row indicates a miRNA. Color gradients indicate the expression level from green (low) to red (high) according to read numbers. miRNAs were declared as differentially expressed if the adjusted treatment *P-value* was <1 × 10^-3^. *P*-values were adjusted to control the false discovery rate using the Benjamini-Hochberg method. Clustering was based on Pearson correlation coefficient and 'average' algorithm. Dis., Disease library; S.m., *Sinorhizobium meliloti*; R.i., *Rhizophagus irregularis*.

To further characterize miRNA regulation during *M. truncatula* root biotic interactions, a statistical analysis of differential accumulation was performed based on the normalized read counts in each library [63] (Additional file 2). First, by comparing miRNAs expressed in roots from plants grown in control conditions (using different experimental settings: soil substrate, hydroponic and aeroponic systems) to those expressed in leaves, we investigated miRNA transcriptional plasticity inside root samples. Leaf miRNA populations are very distinct from those observed in roots (Figure 6a). Indeed, 96 miRNAs (28.2%) were specifically detected in leaves and 47 (14%) in roots (Additional file 6: Figure S6a). Comparison of miRNA abundances in the three 'whole root' control conditions *versus* the triplicate libraries from root tips revealed many miRNAs enriched in root apexes (Figure 6b). In addition, out of 279 miRNAs identified in all those libraries, 28, including 26 novel miRNAs, were only detected in the apex libraries (Additional file 6: Figure S6b). To date, only few miRNAs have known functions in the root apical meristem - *for example,* ath-miR160 involved in root cap differentiation and stem cell niche maintenance in *A. thaliana* and *M. truncatula* [64,65], and mtr-miR396, which restricts root growth and meristematic cell proliferation in *M. truncatula* [28]. Our data suggest that analysis of smRNA populations in root apexes may reveal specific regulation related to post-embryonic root growth and development.

**Figure 7:**
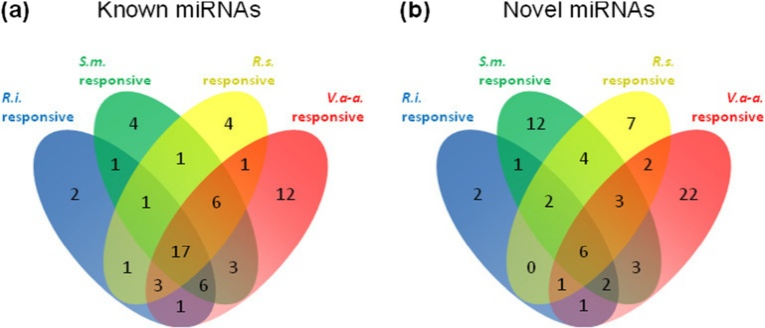
**Venn diagrams of the distribution of differentially expressed miRNAs in response to different symbiotic and pathogenic microorganisms. (a,b)** Venn diagrams of known miRNAs **(a)** and novel miRNAs **(b)**. *R.i.*, *Rhizophagus irregularis*; *S.m., Sinorhizobium meliloti*; *R.s., Ralstonia solanacearum*; *V.a-a., Verticillium albo-atrum.* The numbers represent the number of miRNAs showing a differential response to the microorganism(s) on each Venn diagram area. miRNAs were declared differential if the adjusted treatment *P-value* was <0.01 for R.i., S.m. and R.s., and 1 × 10^-6^ for Va-a. *P*-values were adjusted to control the false discovery rate using the Benjamini-Hochberg method.

To get a global overview of putative miRNA pathways involved in root interactions with pathogenic microorganisms (the bacterium *Ralstonia solanacearum* and the fungus *Verticillium albo-atrum*) or symbionts (the nitrogen-fixing bacterium *Sinorhizobium meliloti* and the mycorrhizal fungus *Rhizophagus irregularis*), the miRNA populations were compared (Additional file 10). Analyses of read abundances in the inoculated root libraries *versus* their respective mock controls revealed a set of 131 miRNAs (31% of the total) potentially differentially expressed under at least one of these interactions. Among them, 23 were regulated in all conditions and, interestingly, 17 of these corresponded to miRNAs reported in miRBase V20 (Figure 7a). On the other hand, most (63.2%) of the novel miRNAs seem to respond preferentially to some of the microorganisms (Figure 7b), notably in response to *V. albo-atrum* (34 miRNAs, 25.9%) and *S. meliloti* (16 miRNAs, 12.2%).

Globally, root inoculation with the symbionts, in contrast to pathogens, mainly led to miRNA induction, suggesting a global repression of their related targets. Both common and specific processes occur during symbiotic and pathogen interactions; *for example,* initial defense responses occur in both cases but, during symbiosis, these responses need to be repressed to allow symbiotic growth and invasion whereas defense must be sustained at the plant level to control pathogen infection [2,5]. The recognition of pathogens or symbionts is under plant host control and the miRNAome data contributed by this study, apart from enhancing our global understanding of *M. truncatula* regulatory pathways, may become useful to define commonalities and differences between pathogen and symbiotic signaling pathways and processes.

### Symbiotic Myc and Nod signals reveal early activation of miRNA regulatory pathways

**Figure 8:**
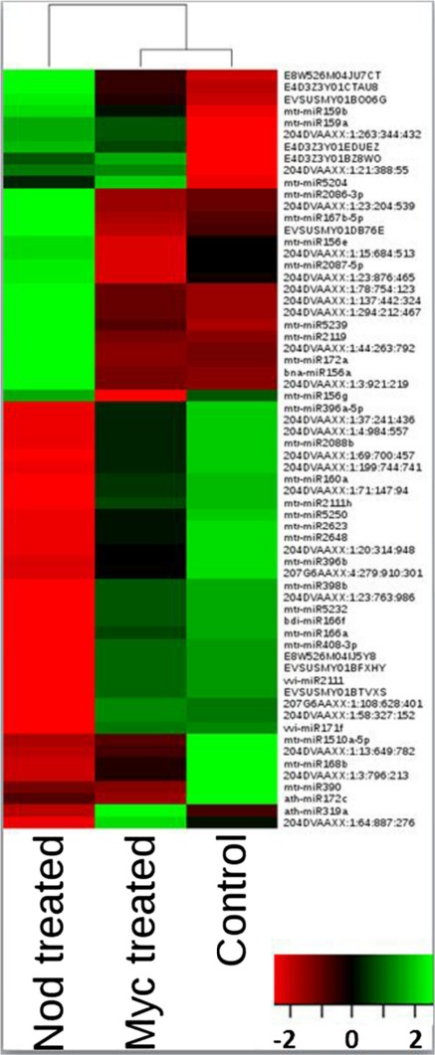
**Heatmap of differentially expressed miRNAs from** ***M. truncatula*** **roots treated with Nod and Myc-LCOs.** Each column corresponds to the different treatments (Nod and Myc-LCO). IDs of differential miRNAs are indicated on the right of the diagram for each row. The tree at the top represents the grouping of the treatments based on the global expression pattern. Color gradients indicate the expression level for each miRNA: green, low; red, high. miRNAs were declared differentially expressed if the treatment *P-value* (adjusted for replication) was <0.05. *P*-values were adjusted to control the false discovery rate using the Benjamini-Hochberg method. Clustering was based on Pearson correlation coefficient and 'average' algorithm.

Plant root endosymbiosis signals essential for the early steps of endosymbiotic interactions have been purified and their biological activity proven using both genetic and biochemical approaches [3]. To decipher miRNA regulatory pathways linked to the initial steps of AM and RL symbiosis, we sequenced smRNA libraries from roots treated or not with purified non-sulfated Myc-LCO and Nod factors. Among the 386 miRNAs detected in these libraries, 62 (16.1%) were differentially expressed (Figure 8; Additional file 10). Strikingly, the expression patterns of the miRNAs responding to Myc-LCO and Nod factors are very different, although these molecules have very similar chemical structures. These results suggest that their perception leads to divergent signal transduction pathways and miRNA regulatory mechanisms specific for each symbiotic interaction. Similar to the results obtained with symbionts, treatment with the signal molecules mostly led to miRNA induction rather than repression. Out of 59 and 27 miRNAs responsive to the Nod or the Myc-LCO treatments, respectively, 35 (59.3%) and 18 (66.6%) were induced, 15 of them by both molecules. In contrast, 20 miRNAs (8 known and 12 novel) were specifically up-regulated after Nod factor treatment (Additional file 6: Figure S6), while only two responded specifically to Myc-LCOs (Additional file 6: Figure S6; Additional file 11). Among the samples from the MtGEA treated with Nod, 29 of the predicted targets show anti-correlated expression with the corresponding miRNAs identified in our study (Additional file 9). These results further support a global miRNA-mediated mechanism of repression of specific targets during symbiotic interactions.

Certain miRNAs were differentially regulated during these processes. For example, three miRNAs (miR156e, miR156g and miR167b) were antagonistically regulated by the two symbiotic signals (induced by Myc-LCO and repressed by Nod signals, respectively). Members of the miR156 family target the SQUAMOSA promoter binding like (SPL) TFs [66,67]. Isoforms of miR156/157 have been found in root apexes and were less abundant in nodules ([48] and this study), which may lead to differential accumulation of the SPL TFs.

Approximately one-third of the miRNAs regulated in the interactions with the two symbionts were also responsive to the corresponding signal molecule (Additional file 6: Figure S7), whereas young miRNAs are more specific to each symbiotic interaction or signal treatment. Genetic and transcriptomic studies revealed many common components in both symbiosis pathways [2], which involve highly similar signaling molecules. However, differences between both pathways exist, such as specific GRAS TFs specialized for one or other symbiotic interaction [68]. The differentially regulated miRNAs identified are good candidates for playing critical roles in the specialization of symbiotic pathways.

### miRNA co-expression analysis identifies functional modules driving the *M. truncatula* root response to biotic interactions and symbiotic signals

**Figure 9:**
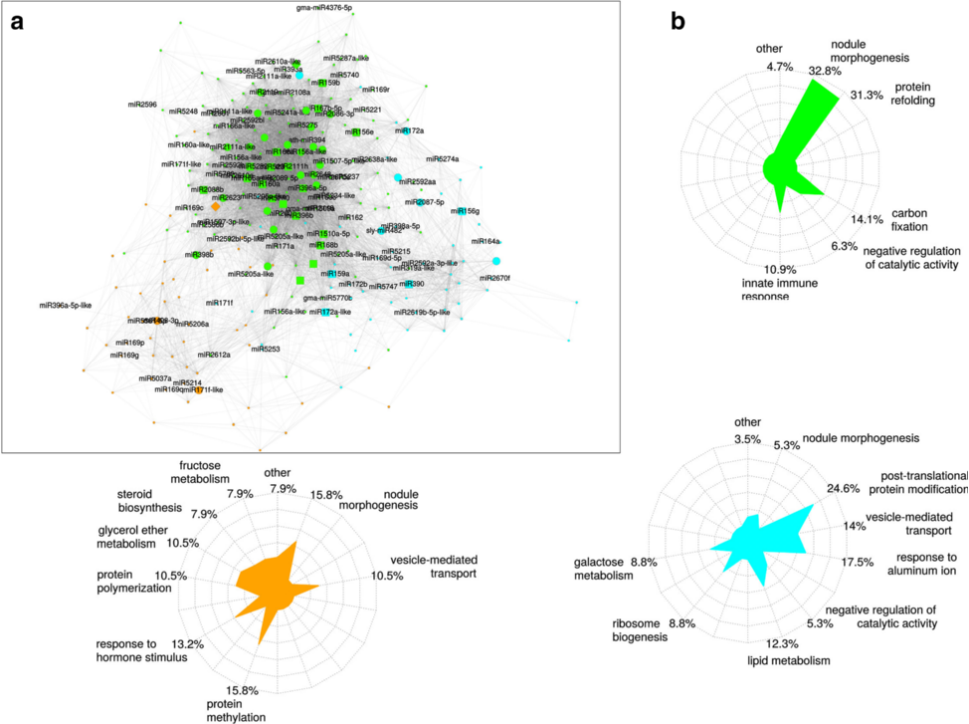
**Weighted gene co-expression network of miRNAs in response to Nod and Myc symbiotic factors. (a)** Different colors (blue, green and orange) represent each regulatory module identified by the WGCNA analysis. The smallest nodes are miRNAs non-responsive to the treatment with the signaling molecules. The biggest nodes are miRNAs responsive to one or both signals. The shape of the node indicates the differential expression pattern: circles represent miRNAs responsive to Nod factor; diamonds represent miRNAs responsive to Myc factor; squares represent miRNAs responsive to both molecules. Names for miRNAs already described in miRBase V20 or new putative variants of known miRNA families are labeled, the latter including the suffix '-like'. Nodes without any label correspond to new miRNAs identified in this study. **(b)** Biological processes associated with the three regulatory modules of miRNA co-expression networks as inferred from the analysis of the Gene Ontology terms of the predicted targets per module. Each radar-plot depicts the repartition of the different biological processes (percentage) for each of the three modules of the network (that is, blue, green and orange). Each branch in the radar plots corresponds to the same biological process.

To identify relevant miRNA-mediated regulatory pathways involved in biotic interactions and symbiotic signals, we investigated the organization of the miRNAomes by applying weighted gene co-expression network analysis (WGCNA) [69]. This correlation network approach identifies groups (modules) of genes with similar expression patterns and high topological overlap [70]. The analysis of miRNA expression data from roots treated with Nod and Myc symbiotic factors and corresponding controls revealed three biologically relevant modules (colored in green, cyan and orange in Figure 9a; another module correlating with experimental replicate variation was not considered for subsequent analyses). The green module comprises 163 miRNAs correlated to the response to Nod factor (r = 0.64), whereas the cyan and orange modules, comprising 61 and 52 miRNAs, respectively, may help in discriminating between Myc and Nod factor miRNA signaling pathways (r = 0.45 and -0.46) (Additional file 12: Table S10a). Differentially conserved miRNAs, already known to be involved in the regulation of symbiotic processes, belong to each module, such as miR164a variant [13], miR166a variant [14], miR169 and miR396. The modules contain also newly discovered miRNAs responsive to single or both signaling factors that are co-expressed with previously validated miRNAs.

To gain further insight into the biological relevance of the mature miRNA regulation network analysis, we analyzed the functional categories of the predicted targets per miRNA module and tested for enrichment of Gene Ontology (GO) terms. The main biological processes associated with each module were shown to be highly different (Chi-squared test *P*-value <2 × 10^-16^). Analysis of the enrichment in GO terms, based on the annotations of the predicted targets of the miRNAs within the different modules (Figure 9b), revealed significantly different patterns of biological processes associated with each module. As a noteworthy example, predicted targets of miRNAs in the green module (correlated with Nod factor response) are mainly involved in nodule morphogenesis (32.8% of the GO terms for annotated predicted targets).

A similar analysis of miRNA expression data was performed on smRNA libraries from root inoculated with symbiotic and pathogenic microbes and their respective controls. Three regulatory modules (differentially colored in green, cyan and orange in Additional file 6: Figure S9a) respectively comprise 150, 46 and 38 miRNAs having correlated expression (Additional file 12: Table S10b). Analysis of the correlations between modules and responses to symbiotic or pathogenic microbes showed that the cyan and green modules appeared to be respectively negatively or positively correlated to the response to *S. meliloti* infection (r = -0.85 and r = 0.64) whereas the orange module grouped miRNAs discriminating symbiotic and pathogenic responses (r = 0.91). Again, certain miRNAs already known to be involved in the regulation of symbiotic and pathogenic processes, such as miR169 variants [20], miR171h and other variants [22,23], miR393 [16], miR396 [15], sly-miR482* and miR2118 [19], were found, suggesting co-regulatory roles for the newly discovered miRNAs present in those modules. Interestingly, analysis of the enrichment of GO terms on target mRNAs (Additional file 6: Figure S9b), showed that miRNAs of the orange module (discriminating symbiotic and pathogenic responses) mainly target genes involved in defense response, proteolysis and nodule morphogenesis (23%, 23% and 12%, respectively). On the other hand, miRNAs of the green module (positively correlated to the response to *S. meliloti* infection) preferentially target genes related to post-translational protein modifications, vesicle-mediated transport and nodule morphogenesis to a lower extent. These functions are in accordance with previous knowledge for symbiotic organogenesis and pathogenic interactions.

Therefore, WGCNA helped us to reveal the structure of miRNA regulation in the *M. truncatula* root in response to pathogenic and symbiotic interactions and signals. Coordinated miRNAome responses were revealed early on when Nod and Myc symbiotic signals are detected by the plant. This response seems associated with significant differences in GO terms for the corresponding targets. The co-expression network approach revealed new and differentially expressed miRNAs grouped in distinct regulatory modules, with known miRNAs modulating plant responses towards symbiotic or pathogenic interactions. Whether or not these new miRNAs are acting coordinately with conserved miRNAs (and are not only co-expressed with them) is of utmost interest to decipher the genome-wide molecular regulation of plant responses to root biotic interactions.

## Conclusions

We have analyzed 20 smRNA libraries covering roots of *M. truncatula* under various conditions, and this large diversity allowed us to identify 52 genuine novel miRNAs, 60 novel variants of already known miRNAs and up to 416 candidate miRNAs expressed in root tissues or linked to the interaction of roots with pathogenic and symbiotic micro-organisms in this model legume.

Polymorphism analyses of 26 ecotypes of *M. truncatula* revealed a strong conservation of both conserved and novel mature miRNA sequences, suggesting purifying selection due to functional constraints. As expected, positions 10 and 11 of miRNAs present a very low SNP rate, indicating that comparative analyses of genotypes provide additional validation of putative miRNA sequences. Interestingly, the conserved miRNAs show an increased number of SNPs in their precursors and flanking regions, a pattern not yet described for other plant species. This suggests differential processing between ecotypes, a sign of balancing selection, which may lead to changes in miRNA-regulated target expression.

Most miRNAs regulated in the early and late stages of bacterial or fungal symbiotic interactions were differentially expressed depending on the interacting symbiont or pathogen, revealing specificities of the root miRNAome for each biotic interaction or symbiotic signal. Network analyses revealed modules of new and conserved co-expressed miRNAs that regulate distinct sets of targets, highlighting potential miRNA-regulated biological pathways relevant to pathogenic and symbiotic interactions. Globally, our study demonstrates the plasticity of the root miRNAome to respond to environmental interactions.

## Materials and methods

### Plant materials

Plants from the reference sequenced line Jemalong A17 or from a *sunn-sickle* hypernodulating double mutant (in the same Jemalong A17 genetic background [25]) of the barrel medic (*Medicago truncatula* L.) were grown in different conditions depending on the interaction of interest. For the interaction with *R. irregularis* (DAOM197198), plants from the A17 line were grown in a greenhouse (16-hour light/8-hour dark cycle) for 7 weeks in a soil substrate inoculated with 1,000 spores/liter. The plants were watered with a Long-Ashton solution with a phosphate concentration of 7.5 μM [23]. Well-established mycorrhization was controlled by the grid intersection method [71]. For the interaction with *S. meliloti,* plants from the *sunn-sickle* hypernodulating double mutant were grown in an aeroponic chamber at 23°C and 75% humidity for 5 days (16-hour light/8-hour dark cycle) in a nitrogenless liquid Fahraeus medium and nebulization inoculated with bacteria. Whole inoculated roots were harvested 5 days after inoculation. For the induction by both Myc-LCO and Nod factors, the A17 plants were obtained in the same way but with Fahraeus medium adjusted to 7.5 μM of phosphate. After 5 days, the plants were transferred in a 10^-8^ M mix of non-sulfated Myc-LCOs (LCO-IV (C16: 0), LCO-IV (C18:1Δ9Z) [3] or Nod factor solution during 4 h or 24 h before whole roots were pooled. Myc-LCO and Nod factors were obtained as described in Maillet *et al.* [3] and Roche *et al.* [72] (for details see Additional file 13). Root tips (1 cm long), were obtained from plants cultivated in a hydroponic culture system on nitrogen-containing Fahräeus medium for 10 days at 23°C and 75% humidity (16-hour light/8-hour dark cycle). For the interaction with root pathogens, seedlings were infected with *R. solanacearum* GMI1000 strain [73] and *V. albo-atrum* V31.2 strain [6] and were grown in a hydroponic culture system on Fahräeus medium (containing nitrate) in a growth chamber with 16 h light at 25°C and 8 h dark at 23°C. Inoculation with *V. albo-atrum* was performed by dipping cut roots of 10-day-old plantlets for 30 minutes in a 10^6^ spores ml^-1^ conidial suspension of the V31.2 strain. Inoculated plants were then transferred back to the nutritive solution and incubated in a growth chamber at 20°C with 16 h photoperiod. For inoculation with *R. solanacearum*, cut roots were immersed for 30 minutes in a bacterial solution of the GMI1000 strain at a concentration of 10^8^ CFU/ml. Inoculated plants and controls treated with water were then placed in a phytotron with 12 h light (170 μmol m^-2^ s^-1^) at 28°C and 12 h dark at 26°C. For each inoculation condition, samples were obtained as pools of roots from 5 to 50 plants harvested every day from one day to one week after inoculation.

### Small RNA isolation and Solexa HiSeq sequencing

Plant roots were harvested and crushed with liquid nitrogen. Total RNAs were extracted using 1 ml of Trizol per 50 to 100 mg of powder. Phenol-chloroform separation, isopropanol precipitation, ethanol wash and RNA solubilization were performed according to the TRI REAGENT manufacturer’s instructions (Ambion, Austin, Texas, USA). For the Myc-LCO and Nod factor libraries and their corresponding controls, total RNAs were isolated from 100 mg of powder with the mirVana miRNA isolation kit (Ambion, Austin, Texas, USA) according to the manufacturer’s instructions. RNA integrity and quality were checked using an Agilent Bioanalyzer 2100.

The smRNA libraries were obtained using the Small RNA Sample Prep kit from Illumina according to the 'Preparing Samples for Small RNA sequencing using the Alternative v1.5' protocol. In brief, a quantity of 5 μg of total RNA per library was used to elute the small RNA fraction (in a size range from 15 nucleotides to 35 nucleotides) from a 12% polyacrylamide-urea gel. Adaptors were ligated to the purified smRNAs at 5’ and 3’ ends and these assemblies were amplified by PCR (12 cycles). Once purified on 6% polyacrylamide gel, the libraries were analyzed using the Agilent Bioanalyzer High Sensitivity DNA and sequenced by the Genoscope (Evry, France) using a HiSeq Solexa sequencer from Illumina. Raw reads for the smRNA sequencing are available in the Gene Expression Omnibus database [74].

### *M. truncatula* genome assembly

Whole genome identification of miRNAs and their targets is highly dependent on the availability of a robust genome sequence. BAC sequencing (Sanger) of the *M. truncatula* A17 genome provided the high quality Mt 3.5.1 genome assembly covering 262 Mbp of the estimated 500 Mbp of the genome [25]. To complement this resource we carried out whole genome shotgun assembly of genome using Illumina technology and a combination of paired-end libraries (0.3 to 0.5 kb inserts, 200× coverage). Data assembly using the SOAPdenovo software (release 1.05) [75] generated 46,525 scaffolds with N50 = 13 kb covering 350 Mbp, including 140 Mbp not present in Mt3.5.1. The combined assembly (260 Mbp Mt3.5.1 + 140 Mbp whole genome shotgun) was annotated using EuGene [76] as described in Young *et al.* [25], and used for miRNA analysis.

### Computational analysis of sequencing data

Raw reads from smRNA sequencing were filtered using the LeARN Pipeline [77] adapted for plant smRNAs. In brief, reads were cleaned (adaptor suppression, 18 to 25 nucleotide size filtering, ‘N’-containing or tRNA and rRNA-like reads suppression) and the redundancy was counted and removed. For smRNA identification, sequences were retained if: (*i*) they matched perfectly at least one but less than 30 times (T. Faraut and E. Courcelle: glint software and source code, http://lipm-bioinfo.toulouse.inra.fr/download/glint/, unpublished) on the *M. truncatula* genome; and (*ii*) they were contained in the top 5% of the most expressed smRNAs of corresponding size in at least one library [28]. Known miRNA sequences (present in miRBase v.20) were also retained. For reads ranging from 20 to 22 nucleotides, a search of a stem-loop secondary structure was performed by extracting the genomic context (400 bp upstream and downstream) surrounding the position of the smRNA sequence and by analyzing those regions with MIRFOLD [78]. Only hairpins fulfilling criteria of plant pre-miRNAs [28,29] were retained (folding energy lower than -30 kcal/mol, folding energy density higher than 0.15, presence of a miR:miR* duplex presenting less than four consecutive mismatches, less than three gaps, and in total less than eight mismatches/gaps).

Sequences of 21, 22, then 20 nucleotides were sorted out in all libraries and considered as 'defining smRNAs' for a particular precursor using a greedy algorithm that scans the list of smRNAs from the highest to the lowest number of reads. For each defining smRNA, the previously detected precursors were then annotated with all sequences ranging from 20 to 24 nucleotides from the libraries. Finally, the defining smRNAs used to annotate the precursor were removed from the list of candidates. The process stopped when all 20- to 22-nucleotide small RNAs had been assigned to a precursor. The selected precursors were classified into five classes depending on their coverage with 20- to 24-nucleotide RNAs. Class 1 precursors only produce smRNAs corresponding to both strands of the miR:miR* region (the region of the stem surrounding the defining miRNA ± 2 nucleotides). Class 2 precursors produce putative miR and miR* as well as additional smRNAs but with lower abundance and outside the miR:miR* region. The mean expression of the additional smRNAs is 10 times smaller than the expression in the miR region. Classes 3 and 4 correspond to classes 1 and 2, respectively, with the difference that no reads for the miR* was found. Finally, for class 5 precursors, smRNAs were more abundant outside the miR region, *that is,* the expression in the miR region was lower than the sum of the reads of all other smRNAs matching on the hairpin. Then, we performed library-specific classifications: for a given library, a precursor was classified if its defining smRNA was contained in the top 5% of the most expressed smRNAs of corresponding size in the library. In parallel, we applied miRDeep-P [30] to these selected precursors. Finally, the positions of all precursors were determined on the latest version of the *M. truncatula* genome (Mt4.0) [79].

A global classification was finally performed following these rules: class n (1 < n < 5) precursors were classified in class n at least in one library. Classes 1 and 2 are considered *bona fide* precursors. For classes 3, 4 and 5, only precursors conserved by miRDeep-P were retained. All these data have been deposited in a dedicated website [31]. Annotation of mature miRNAs and related precursors was performed with NCBI-BLASTN against release 20 of miRBase (June 2013). The variants of newly identified mature miRNAs were clustered into families using CD-HIT (Cluster Database at High Identity with Tolerance) [80] with at least 84.2% identity.

### miRNA target prediction

miRNA targets were predicted *in silico* with miRanda 3.3a [56] modified to consider criteria available for plant miRNA targets and the mRNA from the Mt combined assembly annotation as targets. For this, the penalty score had to be lower or equal to 2 (gap cost = 2; mismatch cost = 1; GU pair cost = 0.5) [81] and the alignment length had to be higher or equal to 18 bp (miRanda parameters: Alignment Score Threshold = 140, Energy Threshold = -20 kcal/mol).

We also predicted miRNA targets using four available *Mt* degradomes (mycorrhizal and non-mycorrhizal *M. truncatula* roots [36]; and mercury-treated or non-treated seedlings [37]). Degradome data were first cleaned (trimmed of adaptor sequences and sequences containing ‘N’ removed). Selected degradome sequences were then perfectly mapped to *M. truncatula* mRNAs with the glint software and then miRNA targets were predicted with Cleaveland 3.0 [58] (option -s 20 read size; use of TargetFinder v1.6 [82]; *P-value =* 0.05). To get additional information, we also used the RNAplex software (v.0.2) [57]. The functional categories of targets were identified using the Gene Ontology Annotation [83]. The GO term classification was performed with CateGOrizer [84] using the Plant_GOslim classification with the 'accumulative all occurrences' count method. Target IDs were also identified by equivalence on the latest version of the *M. truncatula* genome (Mtv4) [79].

To evaluate the miRNA conservation in angiosperms, we searched for homologous sequences of candidates in genomes of six other species: three Fabaceae (*L. japonicus*, *P. vulgaris* and *G. max*), three non-legume eudicots (*V. vinifera*, *P. trichocarpa* and *A. thaliana*) and two monocots (*O. sativa and Z. mays*). smRNA sequences were mapped to the genomes using the glint software (number of mismatches ≤3).

### Polymorphism analysis in *M. truncatula* genotypes and statistical analyses of SNP abundances

We used SNP markers from the *M. truncatula* HapMap project [26] identified by aligning Illumina 90-bp sequence reads from 26 *M. truncatula* accessions to the *M. truncatula* A17 reference genome assembly v.3.5. Those 26 accessions were deeply resequenced up to 30× and represent the overall neutral genetic variability of the species [51]. SNP positions in putative pre-miRNAs, mature miRNAs and 1.5 kb upstream and downstream regions were identified based on gff3 files of pre-miRNAs and mature miRNA positions, using custom awk and bash scripts. Statistical analyses for differential SNP abundances between genome regions and conserved or novel miRNAs were performed using the R software package [85], using a generalized linear model with negative binomial distribution or zero-inflated models for SNPs when required. A significant difference was declared if the *P-value* of the test was <1 × 10^-2^.

### Statistical analyses for differentially expressed miRNAs

Mature miRNA counts were subjected to statistical analyses using the DESeq package [63] of the R/Bioconductor statistical language and customized scripts. Briefly, after adjustment for library sizes, data were modeled by a negative binomial distribution, which allows over-dispersion of counts, and fitted using generalized linear models to test for differential abundances. In the absence of replicates, the 'blind' method was chosen, which conservatively estimates the dispersion for a miRNA by treating measurements between conditions as replicates, based on the observation that most miRNAs are not differentially expressed. In other cases, differential expression is tested in 'maximum' mode, where the dispersion is set to the maximum between the observed dispersion for the miRNA and a fitted curve for the dispersion estimates of all miRNAs. Both of these two procedures are conservative, thus minimizing the number of false-positive differential miRNAs. Logistic regression of the miRNA proportions was also used [86]. *P-value*s were adjusted to control the false discovery rate using the Benjamini-Hochberg method. Venn diagrams in R were generated with the Vennerable package [87].

The anti-correlated expressions of differentially expressed miRNAs and their corresponding targets have been investigated in all the MtGEA samples [88] related to nodulation, mycorrhization, Nod and Myc-LCOs. We selected all the anti-correlated expression values of differentially expressed miRNAs and their corresponding targets that were ≥1.5-fold, between a tested condition and the corresponding control. Target ratio *P-value*s were obtained using Student’s *t*-tests.

### Weighted network construction using WGCNA and GO enrichment analysis

Co-expression networks were constructed using the WGCNA package in R [89] based on normalized read counts for the 416 mature miRNAs. The modules were obtained, after data curation, using the automatic unsigned network construction function with default settings except for a minimum module size of 30 miRNAs, and a threshold for merging modules of 0.25. To maximize the scale free topology fit (R^2^ ≥ 0.8), the beta power was set up at a value of 11 and 29 for miRNA networks in response to symbiotic signals and microorganism interactions, respectively. For each network, the eigengene value was calculated for each module and used to test the association with different linear contrasts of biological interest. For early response to symbiotic chemical signals, these are 'Roots treated with either Nod or Myc factors *versus* Control roots', 'Roots treated with Nod factor *versus* Roots treated with Myc factor', 'Roots treated with Nod factor *versus* control roots', 'Roots treated with Myc factor *versus* control roots' and contrasts accounting for the effect of the three replicates. For responses to microbe comparisons, these are 'Inoculated roots (with either symbionts or pathogens) *versus* Control roots', 'Roots inoculated with pathogens *versus* Roots inoculated with symbionts' and contrasts characterizing the response to each specific microbe (that is, 'Roots inoculated with the microbe *versus* respective mock-inoculated control roots'). The networks were visualized using Cytoscape v3.0.1. [90].

Predicted targets of miRNAs belonging to each module of the networks were functionally categorized based on their GO terms using the REViGO web server [91]. Enrichment in GO terms for targets of miRNAs belonging to each module was tested using Chi-squared tests.

### miRNA quantitative RT-PCR

Total RNAs were isolated with TRIZol reagent (Invitrogen, Carlsbad, New Mexico, USA) and RNA quality and concentration was controlled using a Nanodrop ND1000 spectrophotometer (Thermo Fischer Scientific, Waltham, Massachusetts, USA) and a 2100 Bioanalyzer Instrument (Agilent Technologies, Inc., Santa Clara, California, USA). Real time RT-PCR analysis of smRNAs was performed using the miScript II RT and miScript SYBR Green PCR Kits following the manufacturer's instructions (Qiagen, Venlo, Netherlands) or stem-loop RT-PCR [92]. Total RNA was treated with DNase I, RNase free (Promega, Madison, Wisconsin, USA). Real time PCR was done with ABI 7900HT (Applied BioSystems, Foster City, California, USA) at an annealing temperature of 60°C using specific primers (Additional file 14). Amplification with snU6 specific primers was used as reference (Additional file 14). For all quantitative RT-PCR, two technical replicates were performed for each sample on two independent biological replicates.

## Additional files

Additional file 1: Table S1 smRNA library identifications and corresponding read numbers at the different steps of the pipeline. The first two columns define the libraries and their conditions of production. The other columns list the number of reads selected at each step: reads cleaning, suppression of redundancy, read mapping and filtering by the abundance. The last row pools all the libraries and indicates the read number at each step. Additional file 2: Table S2 Summary table of the properties and conservation of the 416 selected miRNA candidates. **S2a:** miRNAs and variants already present in miRBase. **S2b:** Novel miRNA families accepted by miRDeep (see Methods, genuine miRNAs). **S2c:** potential novel miRNAs not accepted by miRDeep. nt: nucleotides, Mt: *Medicago truncatula*, Lj: *Lotus japonicus,* Gm: *Glycine max*, Vv: *Vitis vinifera*, Pt: *Populus trichocarpa*, At: *Arabidopsis thaliana*, Os: *Oryza sativa.* Green: already registered mature miRNAs or miR*. Yellow: new miRNA variants of already registered mature miRNAs or miR*. Pink: passenger strand sequences present in higher read numbers than their corresponding proposed miRNA (class 3). Grey lines: candidate miRNAs whose genomic loci with less than 20% of structured loci. Additional file 3: Table S3a List of intergenic miRNA precusors. miRDeep tab contains the precursors validated by “miRDeep” software. “miRDeep rejected” tab contains the precursors rejected by the miRDeep software. nt: nucleotide, AS: antisense to the neighboring gene, S: sense to the neighboring gene. Additional file 4: Table S3b List of intragenic miRNA precursors. “miRDeep” tab contains precursors validated by the miRDeep software. “miRDeep rejected” tab contains precursors rejected by the miRDeep software. nt: nucleotide, AS: antisense to the neighboring gene, S: sense to the neighboring gene, CDS: Coding Sequence, UTR: Untranslated Region. Additional file 5: Table S4 52 genuine novel miRNAs. Position column indicates the location of the corresponding mature miRNA in the genome, nt: nucleotide, AS: antisense to the neighboring gene, S: sense to the neighboring gene, CDS: Coding Sequence, UTR: Untranslated Region. “Evolution conservation” column indicates the number of mature miRNA copies present in different plant genomes. Mt: *Medicago truncatula*; Lj: *Lotus japonicus*; Gm: *Glycine max*; Vv: *Vitis vinifera*; Pt: *Populus trichocarpa*; At: *Arabidopsis thaliana*; Os: *Oryza sativa*. Additional file 6: Figure S1 Accumulation of conserved and novel miRNAs. **Figure S2.** Complexity of mtr-miRNA gene families. **Figure S3.** Distribution of the wide-spread miRNA class in the Eudicots, Angiosperms and other plants. **Figure S4.** Comparisons of the levels of polymorphisms of precursors and mature miRNAs from conserved miRNAs encoded in multiple (>2) or simple (<=2) loci. **Figure S5.** Pie charts of the functional annotation (Gene Ontology) distribution of miRNA targets. **Figure S6.** Venn diagrams of the miRNA expression distribution in leaves *versus* roots grown under different conditions. **Figure S7.** Venn diagrams of the miRNA expression distribution in roots treated with symbiotic signals (Nod and Myc LCOs). **Figure S8.** Venn diagrams of the comparison of miRNAs differentially expressed between symbiotic microbes, symbiotic-related signals and respective control roots. **Figure S9.** WGCNA co-expression network of miRNAs in response to root symbionts and pathogens. Additional file 7: Table S5 Polymorphisms data for the 529 pre-miRNAs. Additional file 8: Table S6 Genetic variations in successive 500-bp windows in the 1500-bp upstream and downstream miRNA flanking regions. Additional file 9: Table S7 Expression correlation miRNAs *vs* MtGEA target transcripts. Additional file 10: Table S8 Comparison of miRNA populations between different pathogenic and symbiotic interactions. TPM: transcripts per million. Red and green cells correspond to significantly positive or negative ratio, respectively. Grey cells correspond to *p*-values inferior to 0.01. Additional file 11: Table S9 Differential expression of miRNA in the Nod-LCO and Myc-LCO libraries. TPM: transcripts per million. Red and green cells correspond to significantly positive or negative ratio, respectively. Grey cells correspond to *p*-values inferior to 1.10^−3^. Additional file 12: Table S10 List of mature miRNAs grouped in the different modules of the WGCNA networks. miRNA annotation corresponds to the names for miRNAs already described in miRBase V20 or new putative variants of known miRNA families (−like). **S10a:** List of miRNAs in the WGCNA network in response to Nod and Myc symbiotic factors. **S10b:** List of miRNAs in the WGCNA network in response to root symbionts and pathogens. Additional file 13
**Supplemental methods.**
Additional file 14: Table S11 List of qRT-PCR primers.

## Abbreviations

AM: arbuscular mycorrhizal
CDS: coding sequence
GO: Gene Ontology
LCO: lipo-chitooligosaccharide
miRNA: micro-RNA
MtGEA: *Medicago truncatula* Gene Expression Atlas
nr: non-redundant
RL: rhizobium-legume
siRNA: short-interfering RNA
smRNA: small non-coding RNA
SNP: single nucleotide polymorphism
TF: transcription factor
UTR: untranslated region
WGCNA: weighted gene co-expression network analysis
