# Supplementary material for: The small RNA diversity from Medicago truncatula roots under biotic interactions evidences the environmental plasticity of the miRNAome
Source: Genome Biol. 2014 Sep 24;15(9):457. doi: 10.1186/s13059-014-0457-4 (PMC4212123; doi:10.1186/s13059-014-0457-4)
Supplement: Additional file 6: Figure S1 — Accumulation of conserved and novel miRNAs. Figure S2. Complexity of mtr-miRNA gene families. Figure S3. Distribution of the wide-spread miRNA class in the Eudicots, Angiosperms and other plants. Figure S4. Comparisons of the levels of polymorphisms of precursors and mature miRNAs from conserved miRNAs encoded in multiple (>2) or simple (<=2) loci. Figure S5. Pie charts of the functional annotation (Gene Ontology) distribution of miRNA targets. Figure S6. Venn diagrams of the miRNA expression distribution in leaves versus roots grown under different conditions. Figure S7. Venn diagrams of the miRNA expression distribution in roots treated with symbiotic signals (Nod and Myc LCOs). Figure S8. Venn diagrams of the comparison of miRNAs differentially expressed between symbiotic microbes, symbiotic-related signals and respective control roots. Figure S9. WGCNA co-expression network of miRNAs in response to root symbionts and pathogens. [file 13059_2014_457_MOESM6_ESM.pdf]

Additional file 1: Supplementary figures.

Figure S1: Accumulation of conserved and novel miRNAs

Accumulation of known miRNAs, new variants of already reported miRNA families and novel miRNAs was confirmed by qRT-PCR experiments performed in roots and root tips (a), roots inoculated with *S. meliloti* Sm1021 (b), with *R. solanacearum* (c) or *V. album* (d). To represent small RNA levels, Ct or delta Ct related to snU6 are given (b, c and d). In a) transcript levels in root tips were normalized with roots. (d) Accumulation of known miRNAs, new variants of already reported miRNA families and novel miRNAs in roots inoculated with *V. albo-atrum*. Error bars represent standard deviations. (*) indicates new variants of already reported miRNA families; miR8 to 27 (a), miR-I to III and miR-A to N represent novel miRNAs (see primers in (Additional file 12: Table S10).

Figure S2: Complexity of mtr-miRNA gene families

(a) Distribution of miRNA families according to their gene number. (b) Distribution of miRNA families according to their number of variants. Particular miRNAs mentioned in results are shown in boxes.

Figure S3: Distribution of the wide-spread miRNA class in the Eudicots, Angiosperms and other plants

Numbers represent the total number of miRNAs in each category. Boxes are proportional to the percentage of miRNAs in each category.

Figure S4 : Comparisons of the levels of polymorphisms of precursors and mature miRNAs from conserved miRNAs, that are encoded by multiple (>2) or simple (<=2) locus.

No significant differences (p=0.16) exist for precursor polymorphisms levels among the two precursor classes.

Figure S5: Pie charts of the functional annotation (Gene Ontology) distribution of miRNA targets

Pie charts of (a) conserved miRNA targets; (b) legume miRNA targets and; (c) novel miRNA targets. Each color represents a different functional category and numbers represent the target number belonging to the corresponding category. Targets were predicted as described in methods.

Figure S6: Venn diagrams of the miRNA expression distribution in leaves versus roots grown under different conditions

Venn diagrams of (a) all root libraries vs. leaves; (b) all root libraries vs. root apexes. Numbers on each region of the Venn diagram area represent the amount of miRNA differentially present in each of the different comparisons between conditions or organs. miRNAs were declared differential if adjusted p-value was less than 1.10^{-3} (false discovery rate, Benjamini-Hochberg). R.i.: *Rhizophagus irregularis*; S.m.: *Sinorhizobium meliloti*; Dis.: control library of *Ralstonia solanacearum* and *Verticillium albo-atrum* conditions.

Figure S7: Venn diagrams of the miRNA expression distribution in roots treated with symbiotic signals (Nod and Myc LCOs)
Venn diagrams for (a) known and (b) novel miRNAs. Treatments with Myc-LCOs (Myc) or Nod Factors (Nod) and miRNAs down-regulated (Down) or up-regulated (Up) are indicated. Numbers on each region of the Venn diagram area represent the amount of miRNA differentially present in each of the different comparisons between conditions. miRNAs were declared differential if adjusted treatment p-value was less than 0.05 (false discovery rate, Benjamini-Hochberg).

Figure S8: Venn diagrams of the comparison of miRNAs differentially expressed between symbiotic microbes, symbiotic-related signals and respective control roots
Venn diagrams corresponding to (a) nodulation-related libraries and (b) mycorhization-related libraries. R.i.: *Rhizophagus irregularis*; S.m.: *Sinorhizobium meliloti*. Myc: Myc-LOCs. Nod: Nod factors. Root development: control roots for each symbiotic interaction. Numbers on each region of the Venn diagram area represent the amount of miRNA differentially present in each of the different comparisons between conditions. miRNAs were declared differential if adjusted treatment p-value was less than 1.10^{-3} (false discovery rate, Benjamini-Hochberg).

Figure S9: WGCNA co-expression network of miRNAs in response to root symbionts and pathogens.
(a) Different colors (blue, green and orange) are associated to each regulatory module identified by the WGCNA analysis. Smallest nodes are miRNAs non-responsive to the inoculation with the microbes. Biggest nodes are miRNAs responsive to one or more microbes. The shape of the node indicates differential expression patterns: (○) miRNAs responsive to symbionts, (◊) miRNAs responsive to pathogens, (□) miRNAs responsive to both symbionts and pathogens. Names for miRNAs already described in miRBase V20 or new putative variants of known miRNA families (-like) are labelled. Nodes without any label correspond to new miRNA identified in this study.
(b) Biological processes associated to the three regulatory modules of miR co-expression networks as inferred by the analysis of the GO terms of the predicted targets per module. Each radar-plot depicts the repartition of the different biological processes (%) for each of the 3 modules of the network (i.e. blue, green and orange). Each branch in the radar-plots corresponds to the same biological process.

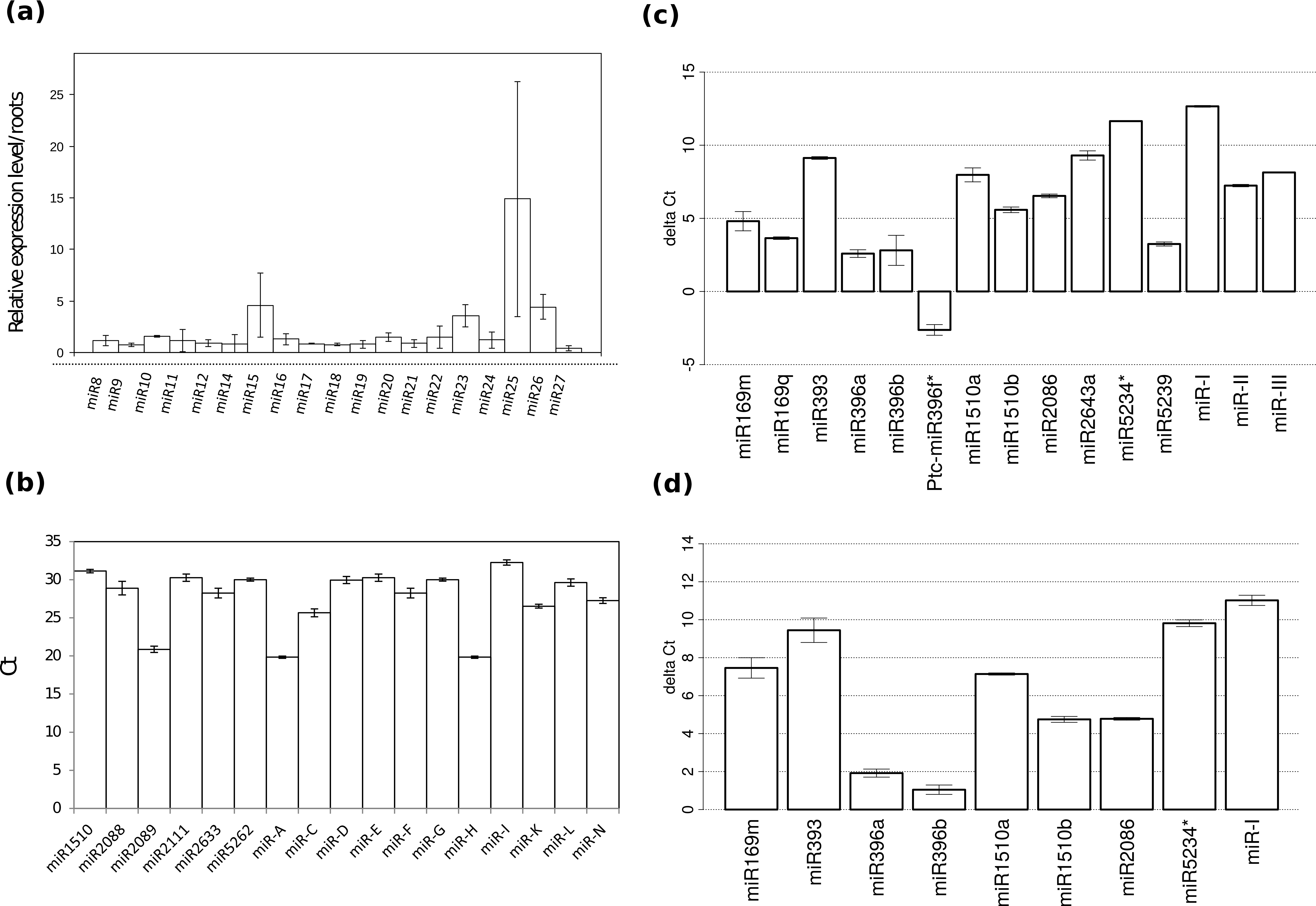
(a)
Relative expression level/roots
0
5
10
15
20
25
miR8
miR9
miR10
miR11
miR12
miR14
miR15
miR16
miR17
miR18
miR19
miR20
miR21
miR22
miR23
miR24
miR25
miR26
miR27
(b)
Ct
0
5
10
15
20
25
30
35
miR1510
miR2088
miR2089
miR2111
miR2633
miR5262
miR-A
miR-C
miR-D
miR-E
miR-F
miR-G
miR-H
miR-I
miR-K
miR-L
miR-N
(c)
delta Ct
-5
0
5
10
15
miR169m
miR169q
miR393
miR396a
miR396b
Ptc-miR396f*
miR1510a
miR1510b
miR2086
miR2643a
miR5234*
miR5239
miR-I
miR-II
miR-III
(d)
delta Ct
0
2
4
6
8
10
12
14
miR169m
miR393
miR396a
miR396b
miR1510a
miR1510b
miR2086
miR5234*
miR-I

Figure S2

(a)

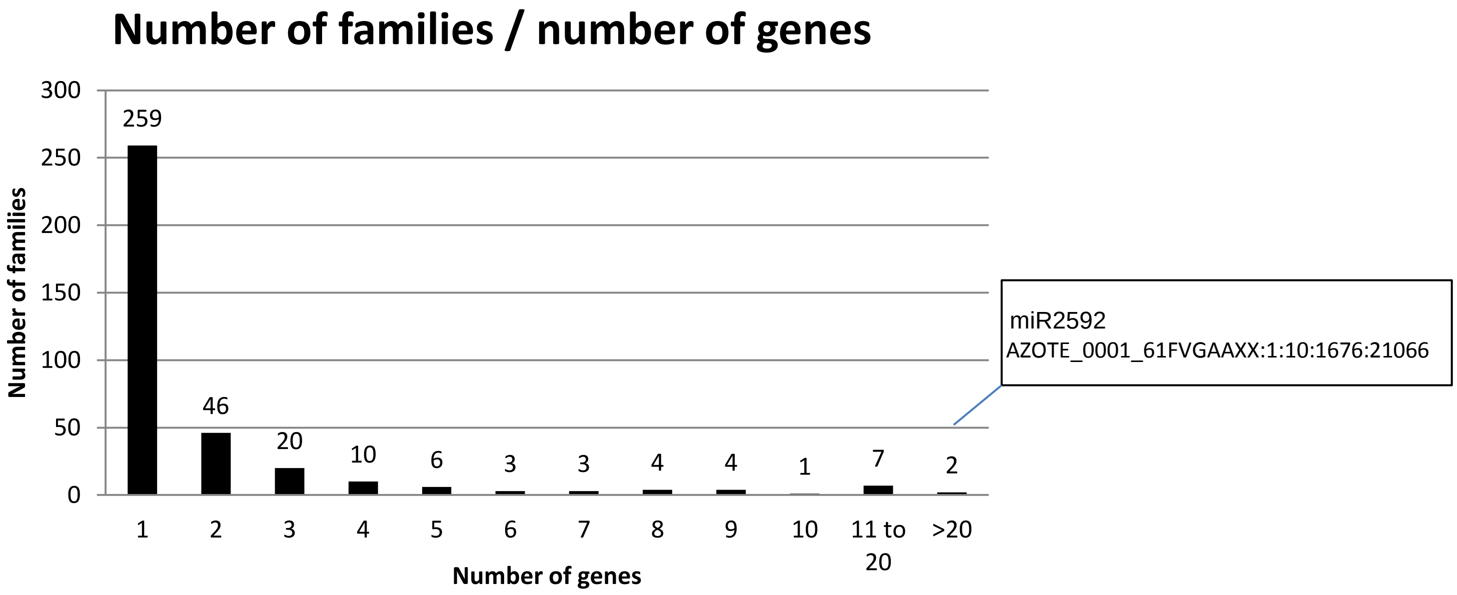
Number of families / number of genes
300
250
200
150
100
50
0
Number of families
259
46
20
10
6
3
3
4
4
1
7
2
1
2
3
4
5
6
7
8
9
10
11 to 20
>20
Number of genes
miR2592
AZOTE_0001_61FVGAAXX:1:10:1676:21066

(b)

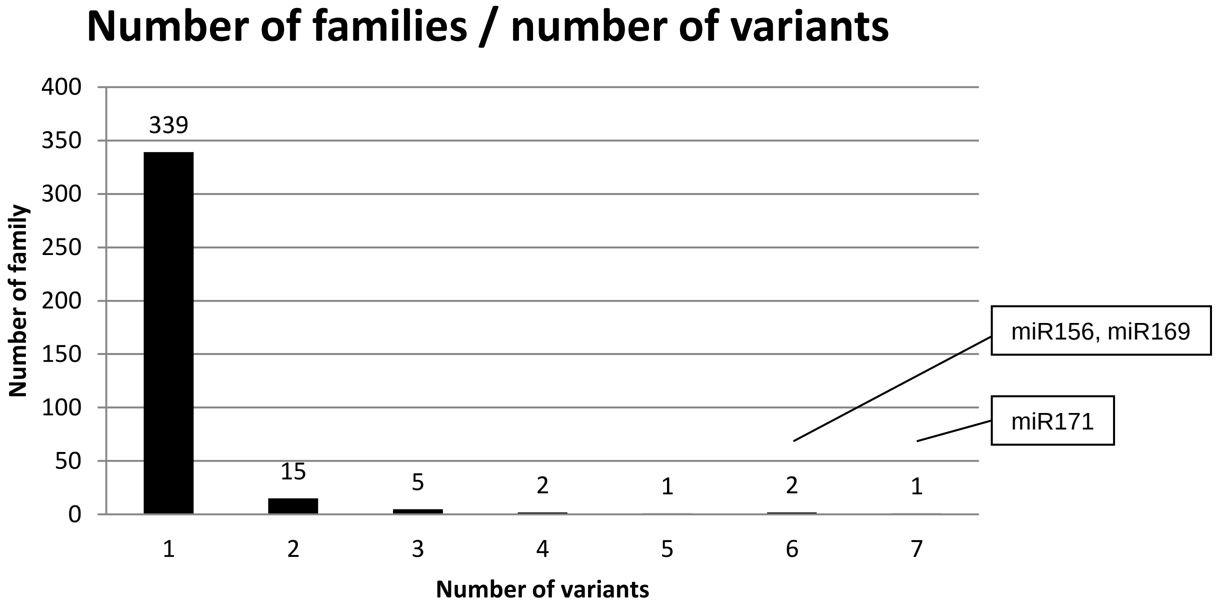
Number of families / number of variants
400
350
300
250
200
150
100
50
0
Number of family
339
15
5
2
1
2
1
1
2
3
4
5
6
7
Number of variants
miR156, miR169
miR171

Figure S3

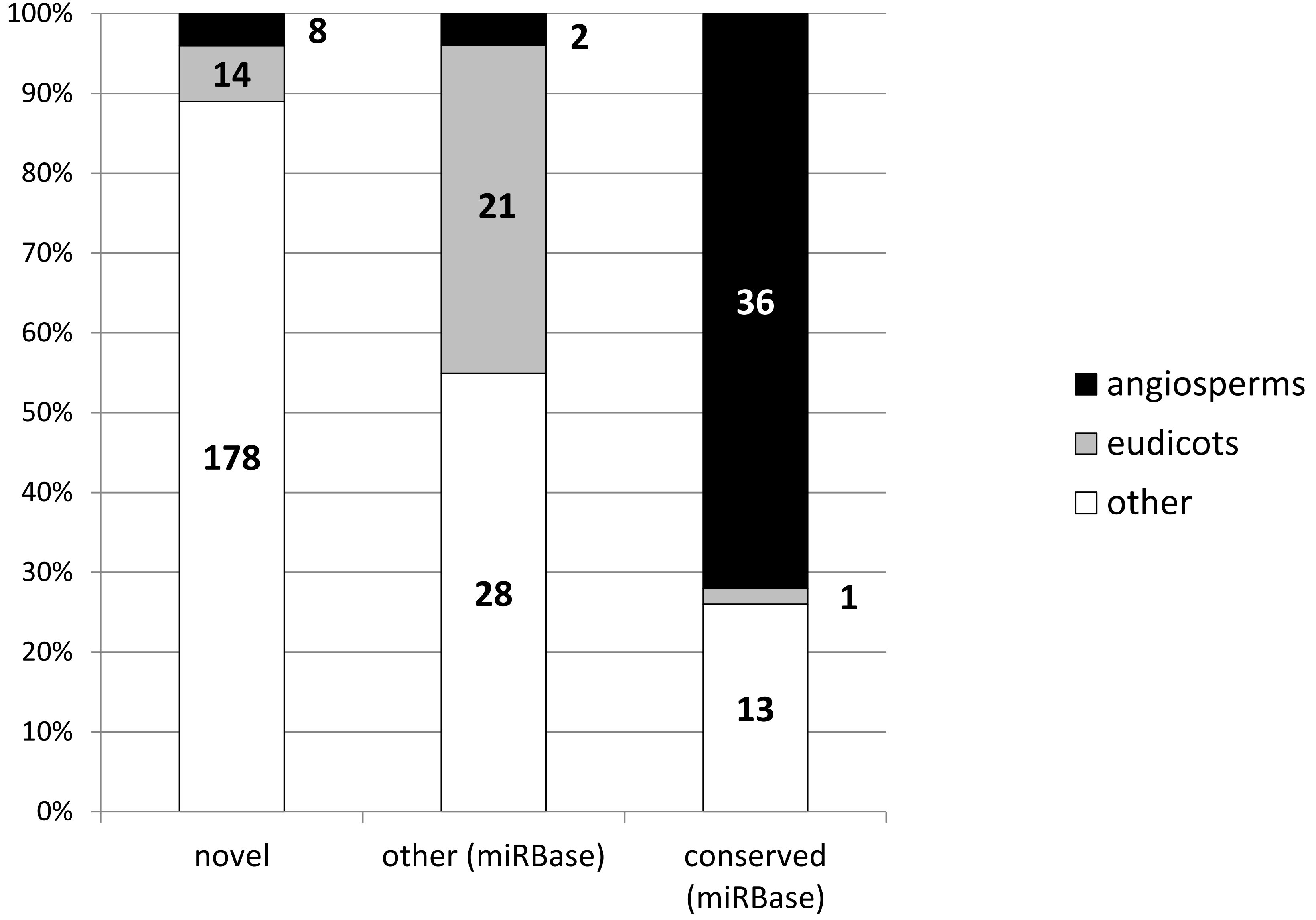

100%
90%
80%
70%
60%
50%
40%
30%
20%
10%
0%
8
14
178
2
21
28
36
1
13
novel
other (miRBase)
conserved (miRBase)
angiosperms
eudicots
other

Figure S4

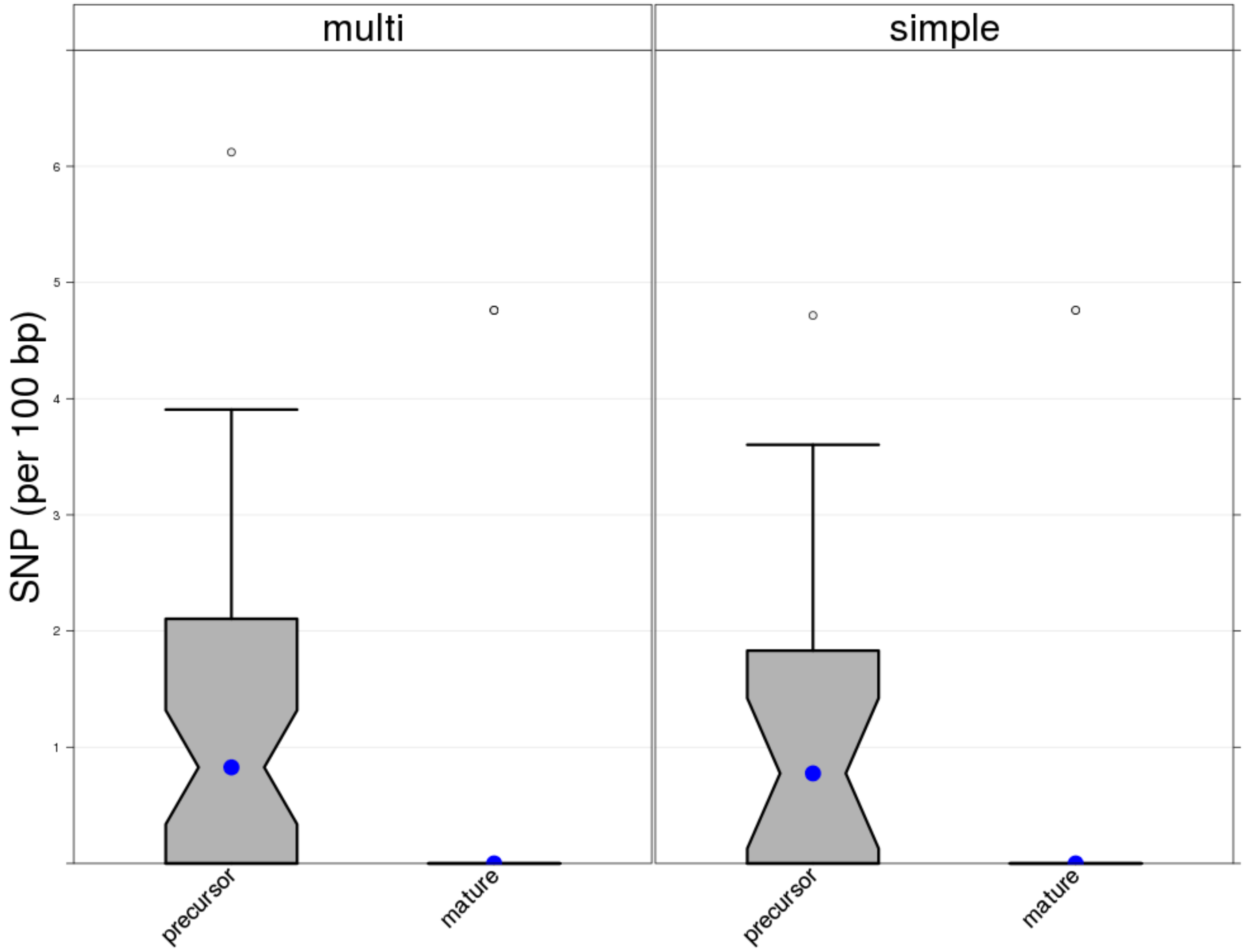

multi
simple
SNP (per 100 bp)
6
5
4
3
2
1
precursor
mature
precursor
mature

Figure S5

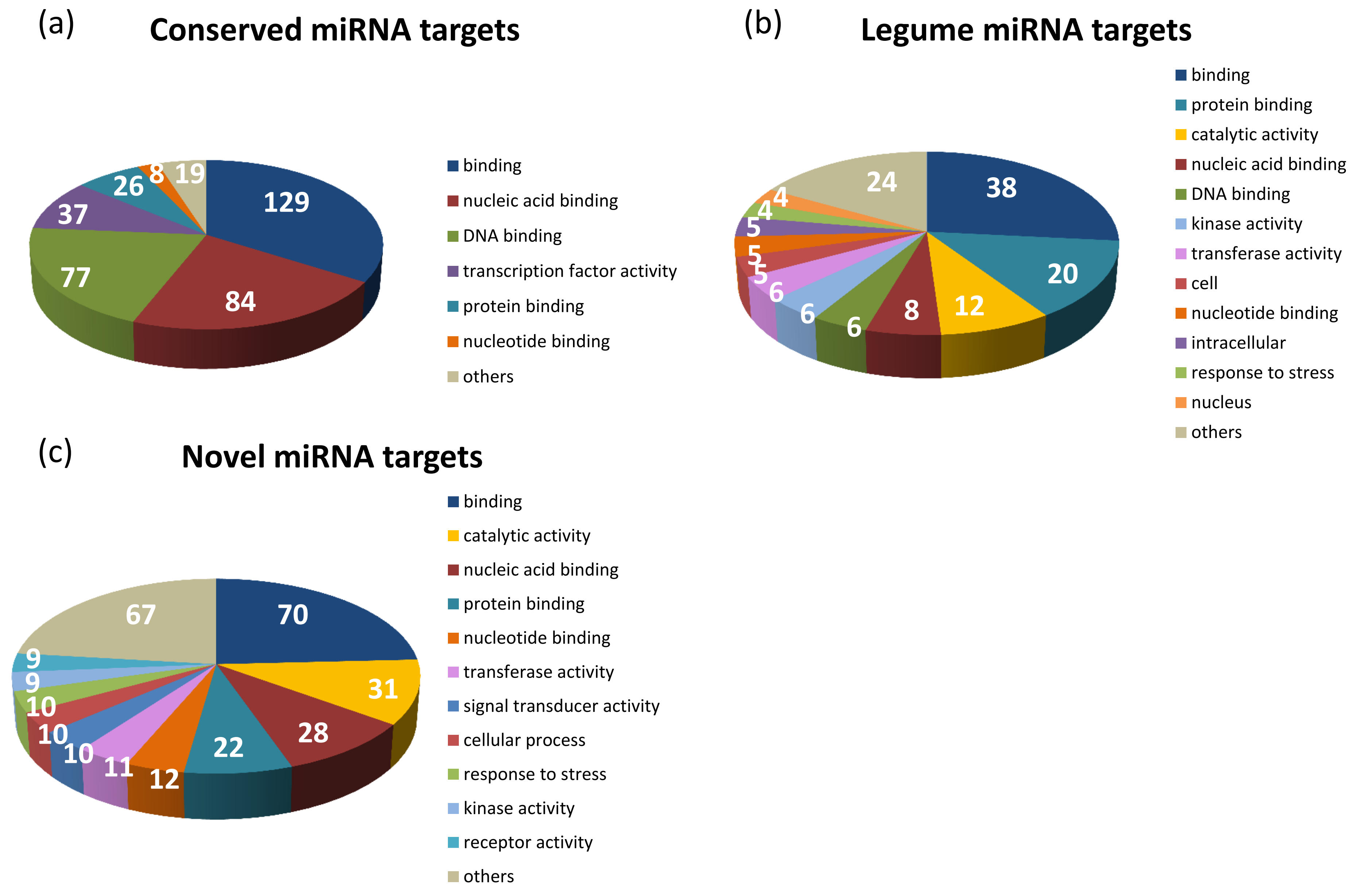
(a)
Conserved miRNA targets
129
84
77
37
26
8
19
binding
nucleic acid binding
DNA binding
transcription factor activity
protein binding
nucleotide binding
others
(b)
Legume miRNA targets
38
20
12
8
6
6
6
5
5
5
4
4
24
binding
protein binding
catalytic activity
nucleic acid binding
DNA binding
kinase activity
transferase activity
cell
nucleotide binding
intracellular
response to stress
nucleus
others
(c)
Novel miRNA targets
70
31
28
22
12
11
10
10
10
9
9
67
binding
catalytic activity
nucleic acid binding
protein binding
nucleotide binding
transferase activity
signal transducer activity
cellular process
response to stress
kinase activity
receptor activity
others

Figure S6

(a)

(b)

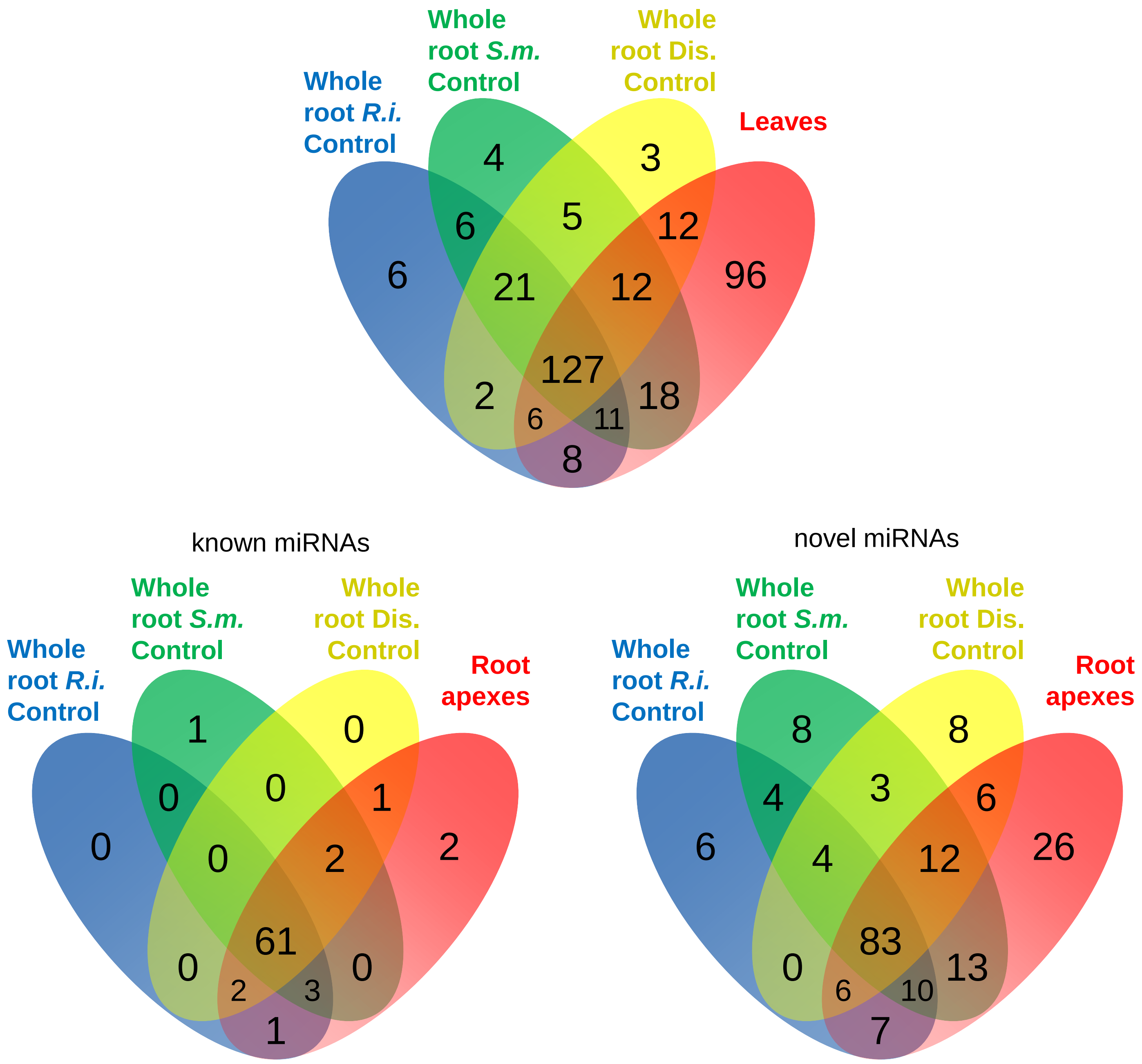
Whole root R.i. Control
Whole root S.m. Control
Whole root Dis. Control
Leaves
6
4
3
96
6
5
12
21
12
127
2
18
6
11
8
known miRNAs
Whole root R.i. Control
Whole root S.m. Control
Whole root Dis. Control
Root apexes
0
1
0
2
0
0
1
0
2
61
0
0
2
3
1
novel miRNAs
Whole root R.i. Control
Whole root S.m. Control
Whole root Dis. Control
Root apexes
6
8
8
26
4
3
6
4
12
83
0
13
6
10
7

Figure S7

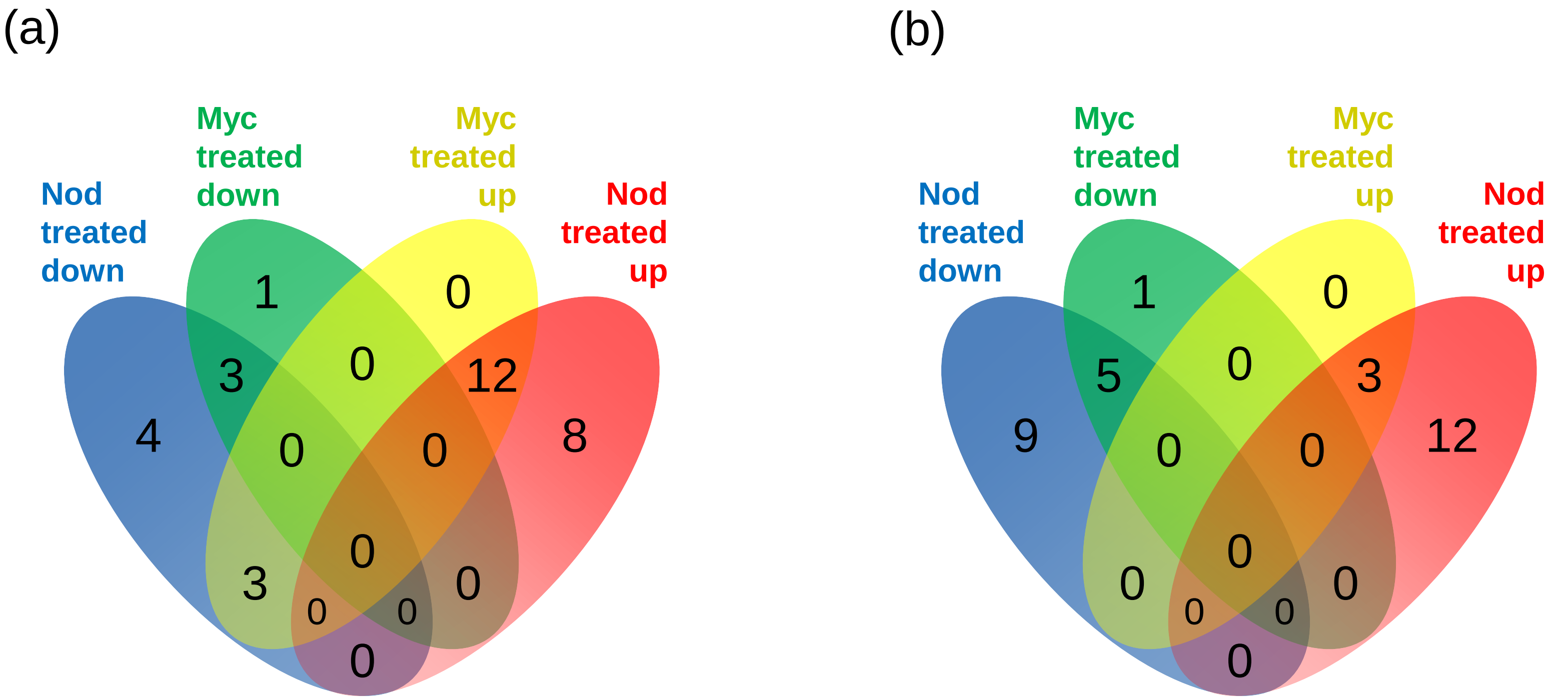
(a)
Nod treated down
Myc treated down
Myc treated up
Nod treated up
1
0
3
0
12
4
0
0
8
0
3
0
0
0
0
(b)
Nod treated down
Myc treated down
Myc treated up
Nod treated up
1
0
5
0
3
9
0
0
12
0
0
0
0
0
0

Figure S8

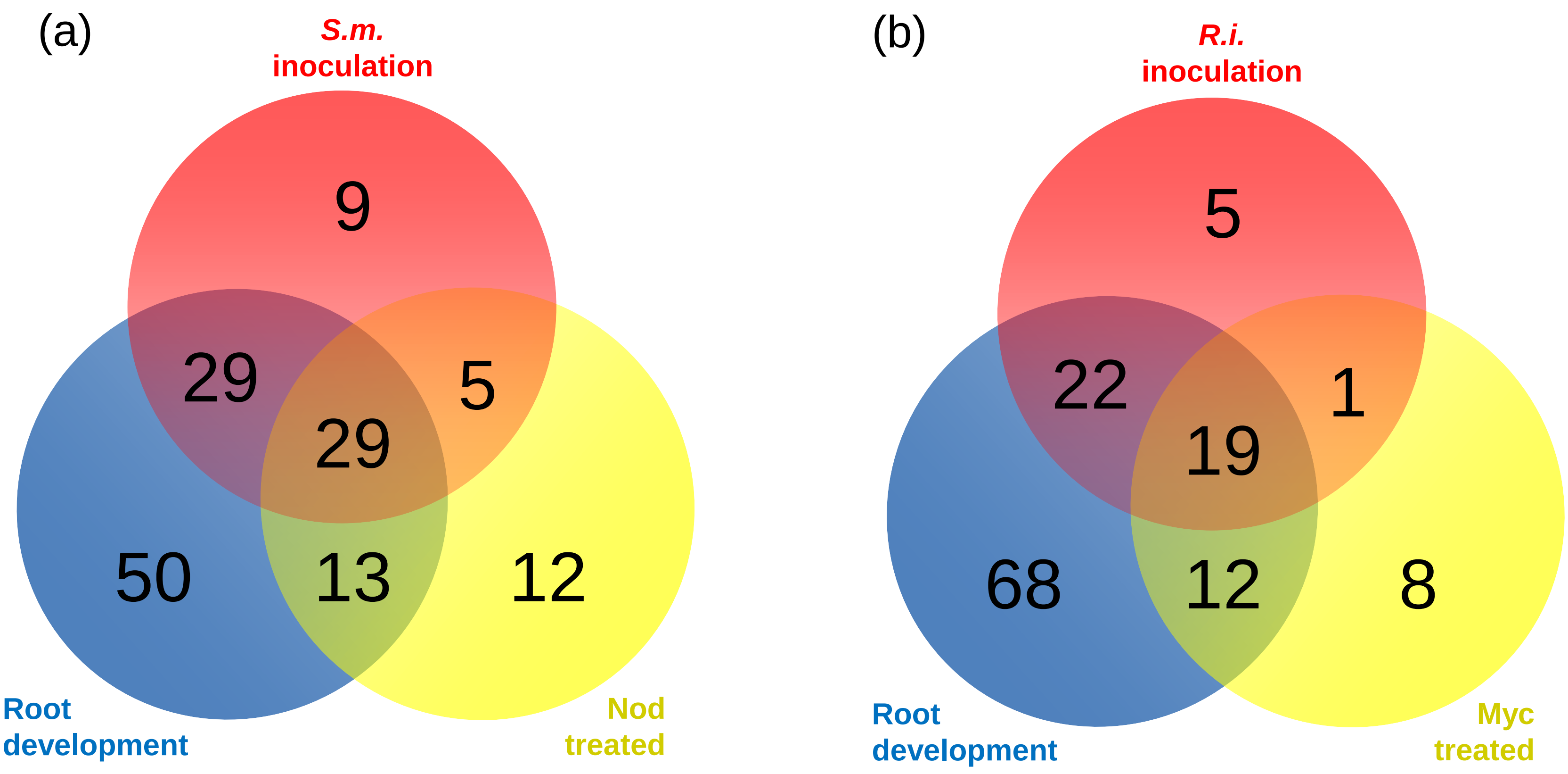
(a)
S.m.
inoculation
9
29
5
29
50
13
12
Root
development
Nod
treated
(b)
R.i.
inoculation
5
22
1
19
68
12
8
Root
development
Myc
treated

Figure S9

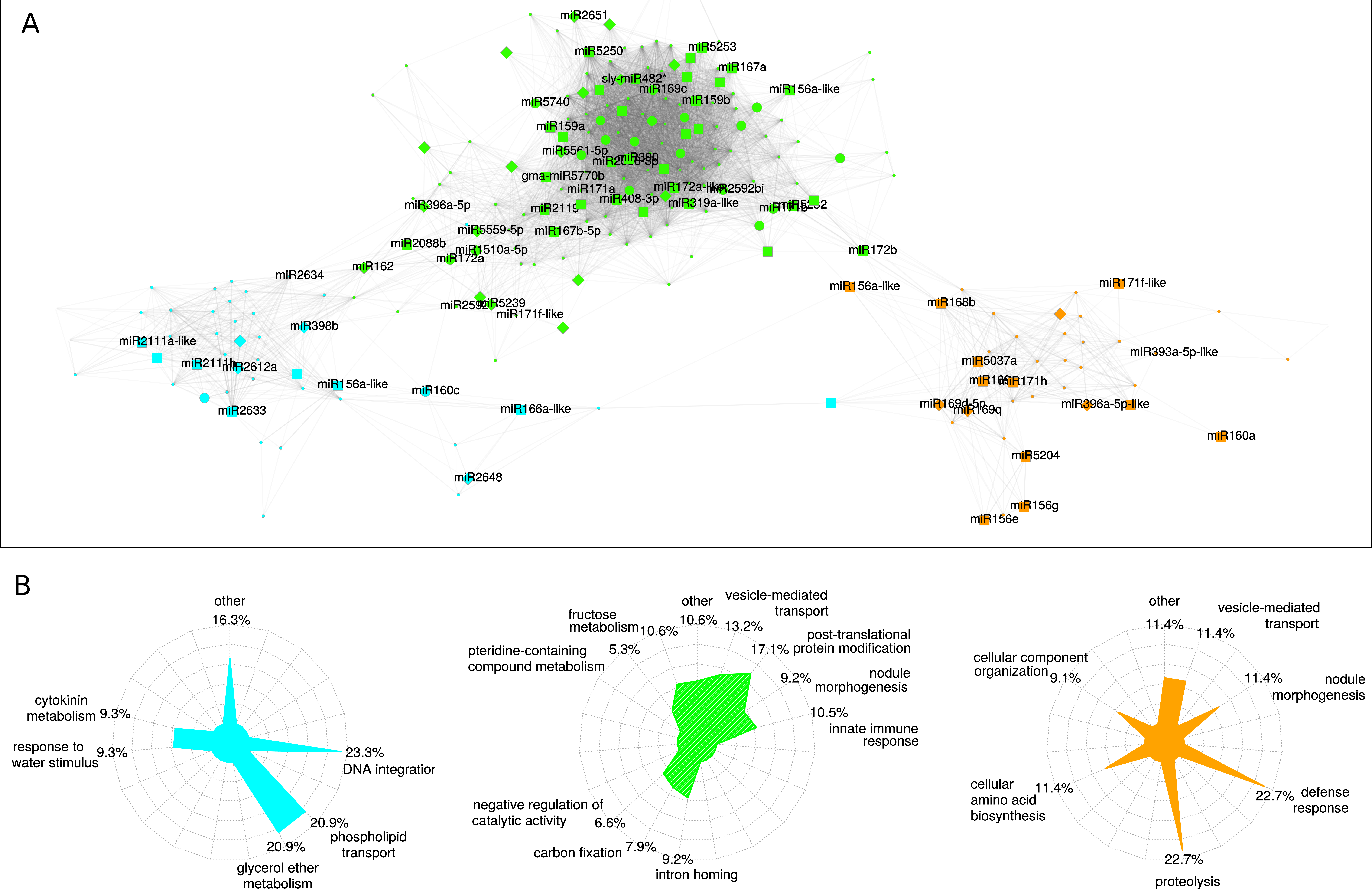
A
miR2651
miR5250
miR5253
miR167a
sly-miR482*
miR169c
miR156a-like
miR5740
miR159b
miR159a
gma-miR5770b
miR171a
miR408-3p
miR319a-like
miR396a-5p
miR5559-5p
miR167b-5p
miR2088b
miR1510a-5p
miR172a
miR172b
miR162
miR2634
miR5239
miR171f-like
miR398b
miR2111a-like
miR2612a
miR156a-like
miR160c
miR2633
miR166a-like
miR2648
miR156a-like
miR171f-like
miR168b
miR393a-5p-like
miR5037a
miR171h
miR169q
miR396a-5p-like
miR160a
miR5204
miR156g
miR156e
B
other
16.3%
cytokinin metabolism 9.3%
response to water stimulus 9.3%
23.3% DNA integration
20.9% phospholipid transport
20.9% glycerol ether metabolism
other 10.6%
vesicle-mediated transport 13.2%
fructose metabolism 10.6%
post-translational protein modification 17.1%
pteridine-containing compound metabolism 5.3%
9.2% nodule morphogenesis
10.5% innate immune response
negative regulation of catalytic activity 6.6%
carbon fixation 7.9%
9.2% intron homing
other
11.4%
vesicle-mediated transport 11.4%
cellular component organization 9.1%
11.4% nodule morphogenesis
cellular amino acid biosynthesis 11.4%
22.7% defense response
22.7% proteolysis
