# Supplementary material for: The small RNA diversity from Medicago truncatula roots under biotic interactions evidences the environmental plasticity of the miRNAome
Source: Genome Biol. 2014 Sep 24;15(9):457. doi: 10.1186/s13059-014-0457-4 (PMC4212123; doi:10.1186/s13059-014-0457-4)
Supplement: Additional file 13 — Supplemental methods. [file 13059_2014_457_MOESM13_ESM.docx]

**Additional file 13: supplemental methods**

**Diffusible factors obtaining**

The Myc-LCOs were obtained as described in Maillet et al. 2011 (Fungal lipochitooligosaccharide symbiotic signals in arbuscular mycorrhizal. Maillet et al. Nature 469, 58–63 (06 January 2011)): Briefly, active LCO compounds were extracted as previously described (Poinsot, V. *et al.* Unusual methyl-branched α,β-unsaturated acyl chain substitutions in the Nod factors of an arctic rhizobium, *Mesorhizobium* sp. strain N33 (*Oxytropis arctobia*). J. Bacteriol. 183, 3721–3728 (2001) ) with the following modifications. No ammonium acetate was added to the eluents and a pre-purification step was used with a solid-phase extraction on a Discovery DSC C18 5-µm (Supelco) column, with three elution steps: 20%, 50% and 100% ACN in water. Samples were collected every minute along the HPLC gradient.

The Nod factors were obtained as described in Roche et al.1991. Briefly, 10 liters of culture medium were extracted twice with 2 liters of butanol. The butanol phase was concentrated to dryness, the residue was then dissolved in 0.5 liter of water, and washed twice with 0.5 liter of ethyl acetate. The aqueous fraction was concentrated and lyophilized. Nod factors were first purified by HPLC on a semipre- parative CIS reverse-phase column (7.5 X250 mm, pBondapak, 10 pm, Waters associates) using a linear gradient from water/ethanol (8020) to ethanol, at a flow-rate of 2 ml/min. The UV absorbance at 220 nm was monitored (Fig. la). The biologically active fraction was then applied to a 1 X 20-cm Sephadex LH-20 column (Pharmacia, Sweden) eluted with ethanol and previously calibrated with polyethyleneglycols. The flow rate was 4 ml/h (Fig. lb). Further purification was done by HPLC on a DEAE column (4.6 X 150 mm; Spherisorb 5 SAX; Biochrom) with a linear gradient from lo-" to 5 X lo-' M ammonium acetate in ethanol, at a flow-rate of 1 ml/min (Fig IC). Final purification was achieved on an analytical CI8 reverse-phase column (4.6 X 250 mm, 5 pm (Spherisorb, Société Francaise de Chromato Colonne) using isocratic conditions (water/acetonitrile (80:20); flow rate, 1 ml/min, UV detection 220 nm, Fig. Id). This last HPLC separation resolved the active fraction into two UV-absorbing peaks. From 4 liters of culture medium of R. meliloti, about 4 mg of purified Nod factors were isolated
